# Targeted genomic sequencing of avian influenza viruses in wetland sediment from wild bird habitats

**DOI:** 10.1128/aem.00842-23

**Published:** 2024-01-23

**Authors:** Kevin S. Kuchinski, Michelle Coombe, Sarah C. Mansour, Gabrielle Angelo P. Cortez, Marzieh Kalhor, Chelsea G. Himsworth, Natalie A. Prystajecky

**Affiliations:** 1 Department of Pathology and Laboratory Medicine, University of British Columbia, Vancouver, British Columbia, Canada; 2 Animal Health Centre, Ministry of Agriculture and Food, Abbotsford, British Columbia, Canada; 3 School of Population and Public Health, University of British Columbia, Vancouver, British Columbia, Canada; 4 Canadian Wildlife Health Cooperative, Abbotsford, British Columbia, Canada; 5 British Columbia Centre for Disease Control, Provincial Health Services Authority, Vancouver, British Columbia, Canada; University of Nebraska-Lincoln, Lincoln, Nebraska, USA

**Keywords:** influenza, genomics, avian influenza, surveillance

## Abstract

Diverse influenza A viruses (IAVs) circulate in wild birds, including highly pathogenic strains that infect poultry and humans. Consequently, surveillance of IAVs in wild birds is a cornerstone of agricultural biosecurity and pandemic preparedness. Surveillance is traditionally done by testing wild birds directly, but obtaining these specimens is labor intensive, detection rates can be low, and sampling is often biased toward certain avian species. As a result, local incursions of dangerous IAVs are rarely detected before outbreaks begin. Testing environmental specimens from wild bird habitats has been proposed as an alternative surveillance strategy. These specimens are thought to contain diverse IAVs deposited by a broad range of avian hosts, including species that are not typically sampled by surveillance programs. To enable this surveillance strategy, we developed a targeted genomic sequencing method for characterizing IAVs in these challenging environmental specimens. It combines custom hybridization probes, unique molecular index-based library construction, and purpose-built bioinformatic tools, allowing IAV genomic material to be enriched and analyzed with single-fragment resolution. We demonstrated our method on 90 sediment specimens from wetlands around Vancouver, Canada. We recovered 2,312 IAV genome fragments originating from all eight IAV genome segments. Eleven hemagglutinin subtypes and nine neuraminidase subtypes were detected, including H5, the current global surveillance priority. Our results demonstrate that targeted genomic sequencing of environmental specimens from wild bird habitats could become a valuable complement to avian influenza surveillance programs.

**IMPORTANCE:**

In this study, we developed genome sequencing tools for characterizing avian influenza viruses in sediment from wild bird habitats. These tools enable an environment-based approach to avian influenza surveillance. This could improve early detection of dangerous strains in local wild birds, allowing poultry producers to better protect their flocks and prevent human exposures to potential pandemic threats. Furthermore, we purposefully developed these methods to contend with viral genomic material that is diluted, fragmented, incomplete, and derived from multiple strains and hosts. These challenges are common to many environmental specimens, making these methods broadly applicable for genomic pathogen surveillance in diverse contexts.

## INTRODUCTION

Avian-origin influenza A viruses (AIVs) pose a perennial threat to poultry and human health. Outbreaks in poultry flocks incur significant economic losses (1, 2). They also expose agricultural workers to novel influenza infections, threatening epidemics and global influenza pandemics (3–5). These outbreaks occur when farmlands become contaminated with excreta from infected wild birds. Numerous wild bird species are naturally infected with diverse AIVs, particularly shore birds and waterfowl (6, 7). These birds live in complex communities, resulting in frequent spillovers between species, reassortment of viral genome segments, and emergence of new strains (8–10). Seasonal migrations along intercontinental flyways allow global dissemination of AIVs (11, 12).

Surveillance of AIVs in wild birds is a cornerstone of outbreak prevention and pandemic preparedness (13–16). Testing is conducted on live-captured birds, hunter-killed birds, and natural deaths recovered from avian habitats. The objective of these surveillance programs is early detection of strains that are pathogenic to poultry and humans. This would allow agricultural producers to increase biosecurity measures and prevent exposure of livestock to infectious excreta. Due to logistical limitations on the number of birds that can be tested, low detection rates, and sampling biases toward certain avian species, these surveillance programs rarely succeed in forewarning the arrival of dangerous AIVs before outbreaks begin in poultry and humans (15).

Alternative AIV surveillance strategies have been proposed wherein environmental specimens from wild bird habitats are tested instead of animals (17, 18). The rationale is that AIVs from diverse members of the wild bird community will accumulate in the environment, including AIVs from avian species that are not commonly tested by surveillance programs. Additionally, environmental specimens are comparatively easy to collect and less disruptive to wildlife. Wetland sediment is one type of environmental specimen in which AIV genomic material has been successfully detected (18–20).

To facilitate AIV surveillance using wetland sediment, we developed a targeted genomic sequencing method to characterize fragments of influenza A virus (IAV) genome in sediment specimens. The method encompasses three components: (i) a custom panel of hybridization probes targeting all IAV subtypes circulating in avian, swine, and human hosts; (ii) sequencing library construction that incorporates a unique molecular index (UMI) on both ends of each genomic fragment in the specimen; and (iii) purpose-built bioinformatic tools that resolve UMIs and allow each distinct fragment of IAV genome recovered to be counted and individually characterized.

The primary purpose of this study was to demonstrate that our targeted genomic sequencing method can recover and subtype IAV genome fragments in real sediment specimens. Since the intended application of these techniques is AIV surveillance, we further characterized fragments from H5 IAVs because lineages within this subtype are currently a global threat to poultry and public health (21). These results show that targeted genomic sequencing of IAVs in wetland sediment is a promising tool for AIV surveillance, particularly early detection of lineages associated with spillover risk in the local wild bird community.

## RESULTS

### Screening sediment for IAV genomic material by RT-qPCR

Four hundred forty sediment specimens were collected from wetlands near Vancouver, British Columbia, Canada, between October 2021 and January 2022. Total RNA was extracted from 435 of these specimens then screened for IAV genomic material by reverse transcription quantitative polymerase chain reaction (RT-qPCR). Seventy-four sediment specimens (17.0%) were positive. An additional 64 specimens (14.7%) were deemed to be suspect positive due to having critical threshold (Ct) values above the cut-off threshold (*n* = 4) or amplification curves trending toward the cut-off threshold in the final cycle (*n* = 60). Sequencing capacity was available for 90 specimens. All 74 positive specimens were assayed. Sixteen randomly chosen suspect-positive specimens were also assayed to assess whether sequencing specimens with indeterminate RT-qPCR results would be worthwhile during future surveillance efforts.

### Detection of IAV genome fragments in sediment by probe capture-based targeted genomic sequencing

IAV genome fragments in these specimens were captured and enriched using a custom panel of hybridization probes. The panel was designed for One Health IAV surveillance, targeting all segments of the IAV genome and providing broadly inclusive coverage of all subtypes circulating in avian, swine, and human hosts (Tables S1 and S2). Captured material was sequenced (Table S3) and then analyzed with two purpose-built bioinformatic tools called HopDropper and FindFlu. HopDropper uses UMI-based analysis to generate consensus sequences for each distinct fragment of IAV genome recovered (22). FindFlu characterizes these fragment consensus sequences and determines the IAV genome segments from which they originated.

**Fig 1:**
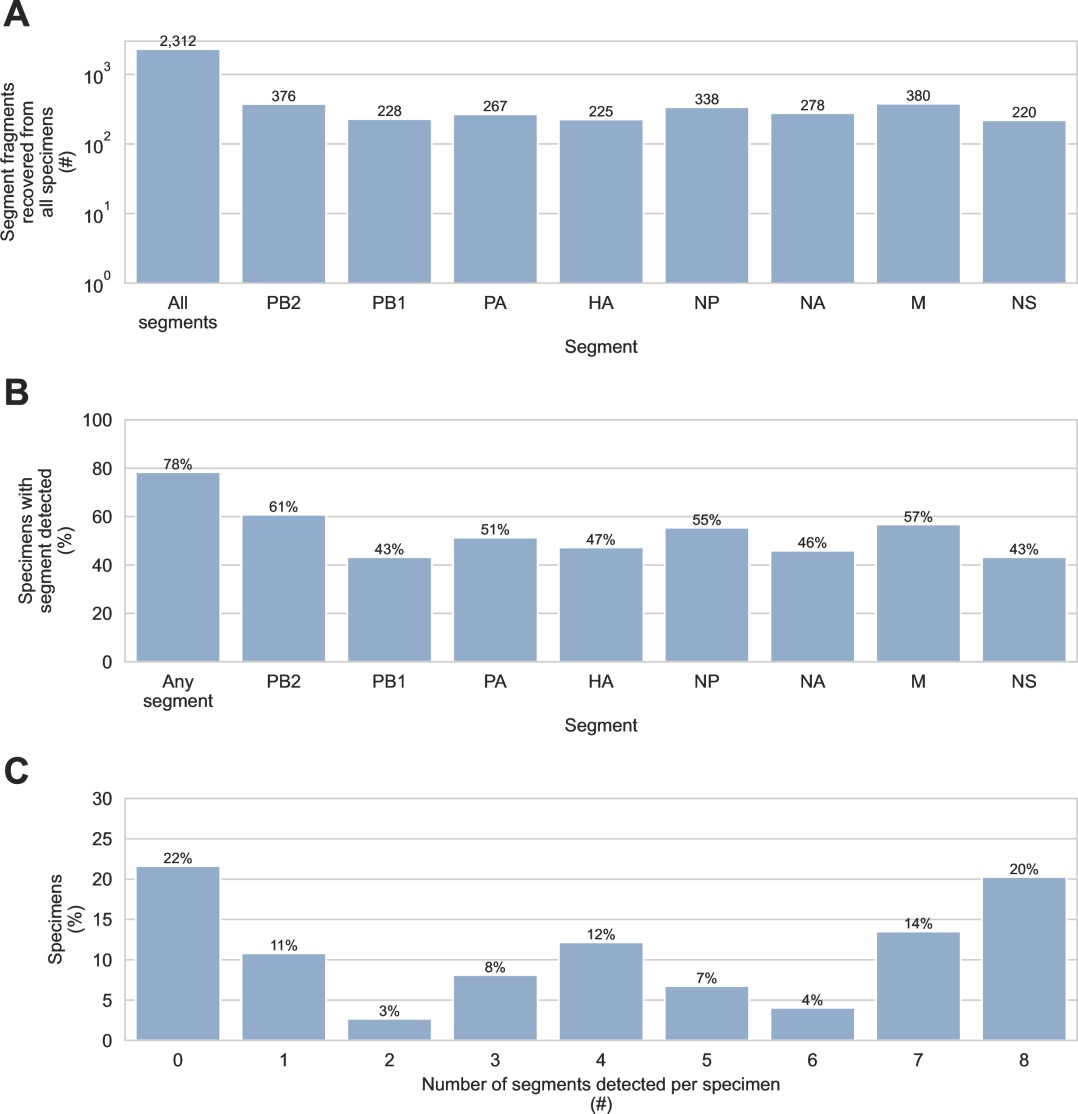
Detection of influenza A virus genome fragments in sediment by probe capture-based targeted genomic sequencing. IAV genome fragments were recovered using probe capture-based sequencing from 74 sediment specimens that had previously tested positive for IAV genomic material by RT-qPCR. (**A**) The number of IAV genome fragments recovered from all specimens was counted. In addition to the total count, the number of fragments originating from each of the 8 IAV genome segments (PB2, PB1, PA, HA, NP, NA, M, and NS) was also determined. (**B**) The sensitivity of probe capture-based targeted genomic sequencing was determined for specimens that tested positive by RT-qPCR. Overall sensitivity was calculated as the percentage of specimens positive by RT-qPCR where probe capture-based targeted genomic sequencing detected at least one IAV genome fragment from any genome segment. Sensitivity was also calculated for each of the IAV genome segments separately. (**C**) The number of different IAV genome segments detected in each specimen was determined.

We detected 2,312 IAV fragments in specimens that were positive by RT-qPCR (Fig. 1A). Only eight IAV fragments were detected in suspect-positive specimens. Low recovery from specimens with indeterminate RT-qPCR results indicated that future surveillance activities should focus on positive specimens only. To reflect surveillance based only on specimens positive by RT-qPCR, the 16 suspect-positive specimens and the 8 IAV genome fragments recovered from them were omitted from the following analyses.

**Fig 2:**
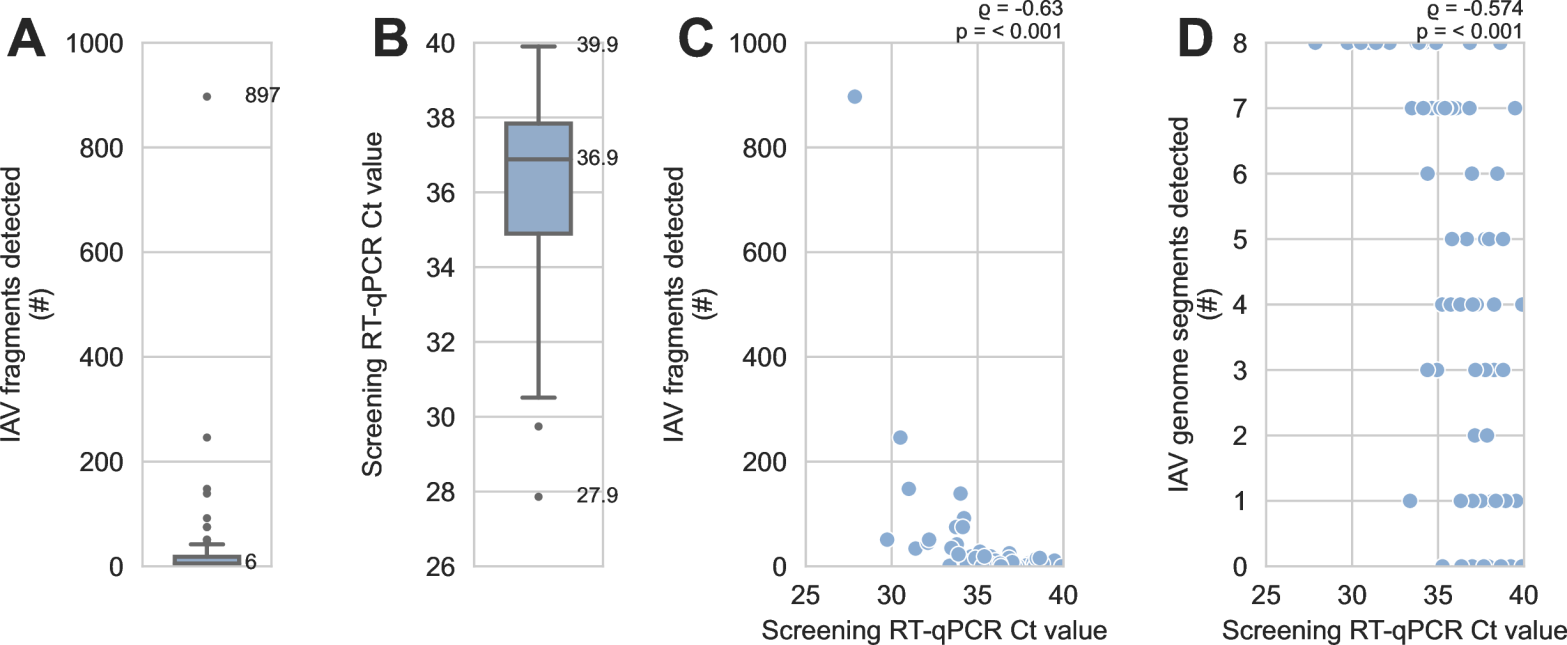
Detection of influenza A virus genome fragments was limited by low abundance of viral genomic material in sediment specimens. A total of 2,312 fragments of IAV genome were recovered using probe capture-based sequencing from 74 sediment specimens that had previously tested positive for IAV genomic material by RT-qPCR. (**A**) The number of IAV genome fragments recovered per specimen was counted. This distribution includes specimens where no IAV fragments were recovered. The median and maximum are indicated. (**B**) Distribution of screening RT-qPCR Ct values for specimens, including specimens where no IAV fragments were recovered. The minimum, median, and maximum are indicated. (**C**) There was a moderate and statistically significant monotonic association between screening RT-qPCR Ct values and the number of IAV genome fragments detected by probe capture-based targeted genomic sequencing. Results of Spearman’s rank correlation are indicated above the upper-right corner of the scatterplot. (**D**) There was a moderate and statistically significant monotonic association between screening RT-qPCR Ct values and the number of different IAV genome segments detected by probe capture-based targeted genomic sequencing. Results of Spearman’s rank correlation are indicated above the upper-right corner of the scatterplot.

IAV genomic material was detected by probe capture-based sequencing in 58 of 74 specimens (78%) that tested positive by RT-qPCR, and fragments from all 8 IAV genome segments were recovered (Fig. 1A and B). IAV fragments were not evenly distributed across specimens, however. The three specimens with the most IAV fragments contained 897, 246, and 148 IAV fragments, respectively. Collectively, these three specimens yielded 56% of all IAV fragments detected. The median specimen contained only six IAV fragments (Fig. 2A). Furthermore, only 20% of specimens contained fragments from all eight genome segments (Fig. 1C), and no individual genome segment was detected in more than 61% of specimens (Fig. 1B).

These results, together with the high Ct values observed when screening specimens by RT-qPCR (Fig. 2B), suggested that low abundance of viral material in these specimens caused stochastic recovery of incomplete genomes by probe capture. Indeed, there was a statistically significant monotonic association between lower Ct values (i.e., greater abundance of viral genomic material) and higher numbers of IAV fragments detected (Fig. 2C). Lower Ct values were also significantly associated with the detection of more of the IAV genome segments (Fig. 2D).

### Diversity of IAV subtypes detected

**Fig 3:**
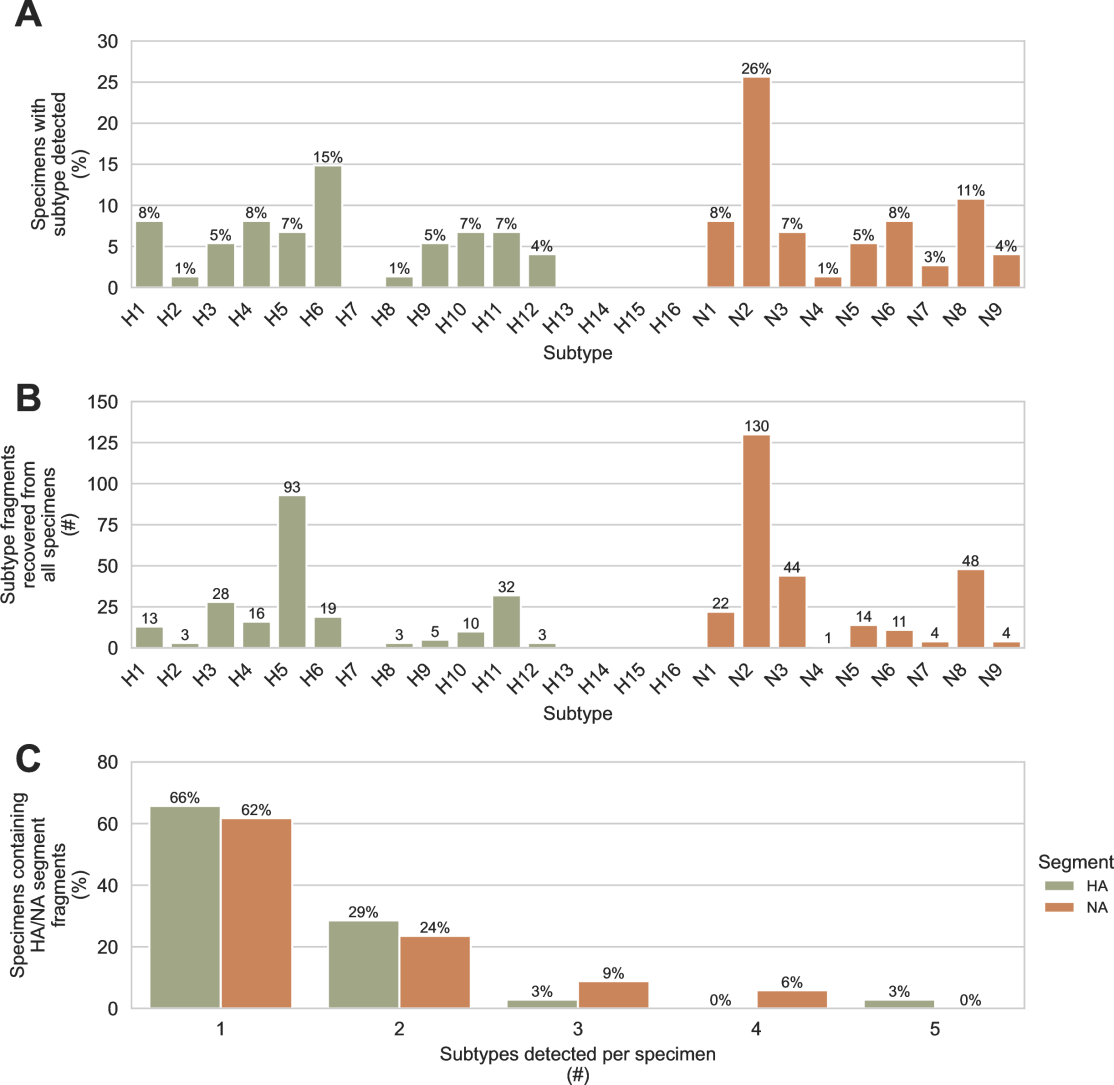
Diverse hemagglutinin and neuraminidase subtypes were detected in wetland sediment using probe capture-based targeted genomic sequencing. Haemagglutinin (HA) and neuraminidase (NA) genome segment fragments were recovered using probe capture-based sequencing from 74 sediment specimens that previously tested positive for IAV genomic material by RT-qPCR. A total of 225 HA fragments were recovered from 35 specimens, and 278 NA fragments were recovered from 34 specimens. (**A**) The percentage of specimens containing each HA and NA subtype was determined. (**B**) The total number of HA and NA fragments recovered for each HA and NA subtype was counted. (**C**) The number of different HA subtypes detected in each HA-positive specimen was determined, and the number of different NA subtypes detected in each NA-positive specimen was determined.

Subtyping the hemagglutinin (HA) and neuraminidase (NA) genome segments is central to IAV surveillance and diagnosis, so our bioinformatic tool FindFlu automatically reports the subtypes of all HA and NA fragments identified. We observed a high diversity of HA and NA subtypes in the 74 sediment specimens that tested positive for IAV material by RT-qPCR. Of the 16 avian-origin HA subtypes, 11 were detected, and all 9 of the avian-origin NA subtypes were detected (Fig. 3A and B). One of the proposed advantages of using environmental specimens for surveillance is the possibility that individual specimens might contain diverse viruses deposited by multiple hosts. We assessed this by counting the number of different HA or NA subtypes present in the same specimen (Fig. 3C). Up to five different HA or NA subtypes were observed in the same specimen. Thirty-four percent of HA-positive specimens contained more than one HA subtype, and 38% of NA-positive specimens contained more than one NA subtype.

### Assessing confidence in detections based on limited numbers of recovered genome fragments

**Fig 4:**
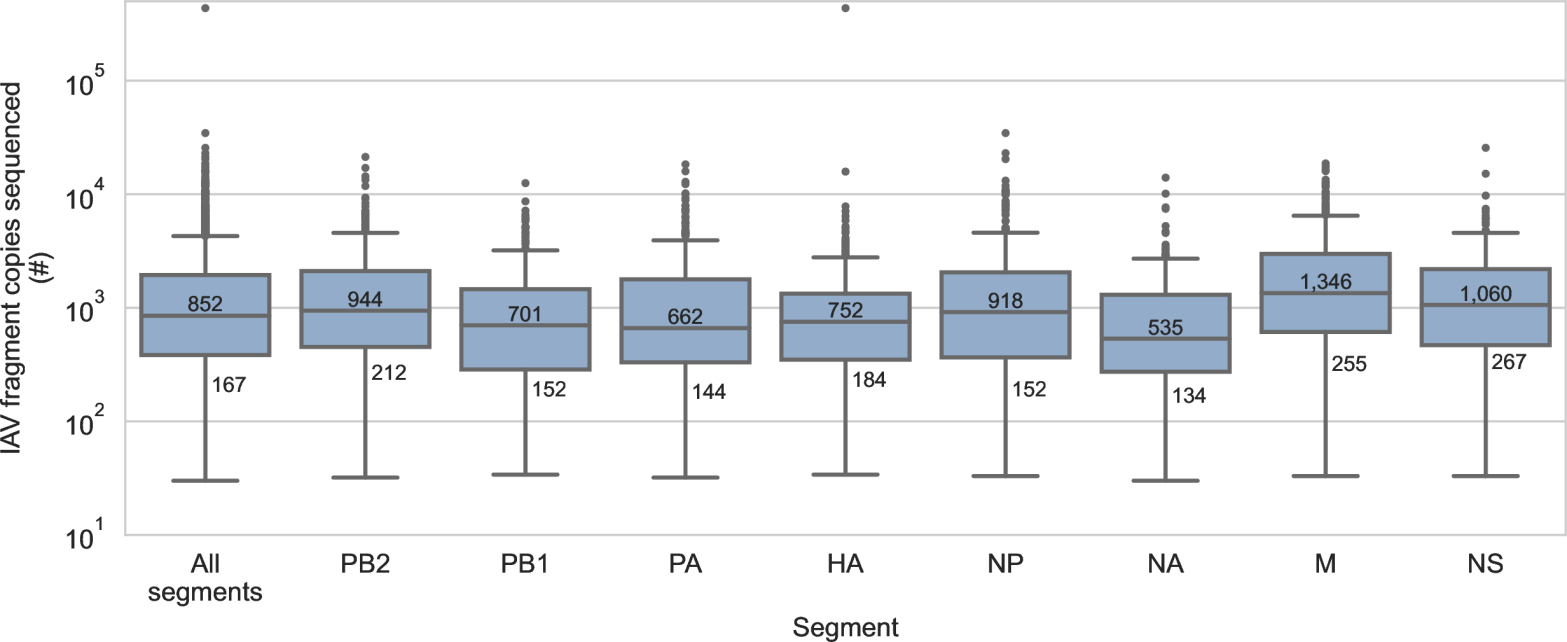
Recovered fragments of influenza A virus genome were sequenced deeply. IAV genome fragments were recovered using probe capture-based sequencing from 74 sediment specimens that previously tested positive for IAV genomic material by RT-qPCR. Multiple copies of each IAV fragment were sequenced, increasing sequencing depth per fragment. The median and 10th percentile of copies sequenced per fragment are indicated overall and for each IAV genome segment.

Many of the segment/subtype detections in this study were based on the presence of a limited number of fragments (Fig. 2A; Fig. S1). This suggested the possibility of false detections. First, we considered whether some of these detections were caused by demultiplexing artefacts, e.g., mutations or base calling errors in library barcodes that occasionally caused limited numbers of IAV reads to be misassigned to incorrect libraries. To assess this, we determined the number of read pairs that described each IAV fragment (Fig. 4). The median number of read pairs per IAV fragment was 852, and 90% of all IAV fragments were described by at least 167 read pairs. Based on these high read pair counts, the actual presence of these IAV fragments in their assigned libraries was strongly supported.

Next, we considered cross-contamination of IAV genomic material between specimens during laboratory handling as a source of false detections. When designing this custom targeted genomic sequencing method, we anticipated the potential for cross-contamination between specimens and incorporated strategies to mitigate this risk. First, the positive control target for this method is a synthetic oligomer with an artificial, computer-generated sequence that does not resemble IAV or any other organism. This ensures that positive controls cannot contaminate surveillance specimens with exogenous IAV genomic material. Second, negative controls are composed of commercially prepared human reference RNA background material. Unlike typical water blanks, these contain sufficient total RNA mass for robust library construction, thereby providing more sensitive detection of low-level cross-contamination. No IAV fragments were observed in any of the six negative controls processed alongside sediment specimens in this study (Table S3). Taken together, this method design and these control specimen results indicated that cross-contamination was not a measurable source of false detections in this study.

Finally, we considered whether index hopping had attributed detections to incorrect libraries. Index hopping is a form of chimeric PCR artefact where library molecules acquire the barcodes of another library during pooled amplification reactions (23–25). We anticipated index hopping during the post-capture PCR step of this method for three reasons. First, libraries are pooled for capture, so a variety of library barcodes are present on template molecules in the post-capture PCR. Second, the low abundance of viral genomic material in these libraries requires extensive amplification during the post-capture PCR. Third, the post-capture PCR provides favorable conditions for chimera formation because of the numerous amplification cycles, low abundance and complexity of captured material, and fragmented condition of viral genomes.

To identify index hops and other chimeric artefacts, we adopted library construction techniques that associate a UMI with both ends of each genomic fragment. This was combined with paired end sequencing on captured material to identify the pair of UMIs associated with each sequenced molecule. A purpose-built bioinformatic tool called HopDropper, which analyzes the frequency and co-occurrences of UMIs, was used to discard sequencing reads originating from potential chimeras and index hops. To confirm the removal of chimeras and index hops by HopDropper, we performed two independent probe captures on the pool of libraries prepared from these specimens; then, we separately analyzed each capture with HopDropper. We reasoned that UMIs enriched by both captures should de-multiplex to the same library and be paired with the same other UMI after each capture. A total of 2,191 UMIs were enriched in both replicates. Of these UMIs, 2,172 (99.1%) were de-multiplexed to the same library in both replicates, and 2,148 (98.0%) were paired with the same other UMI in both replicates. This indicated that chimeric artefacts formed during post-capture PCR were largely absent following analysis by HopDropper and that index hopping was not responsible for systematic false IAV detections in this study.

### Length of IAV fragments recovered by probe capture-based targeted genomic sequencing

**Fig 5:**
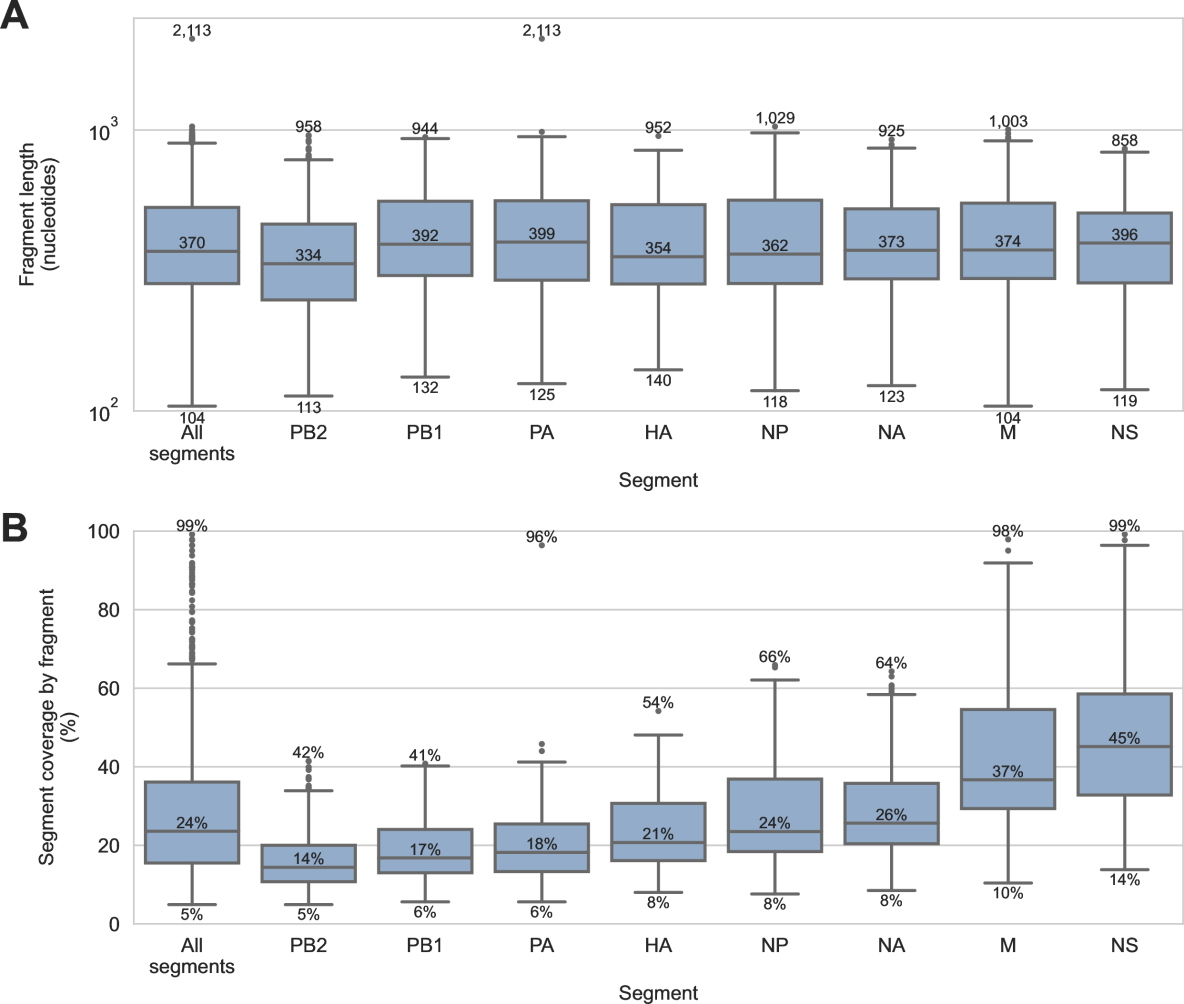
Length of influenza A virus genome fragments recovered from sediment specimens by probe capture-based targeted genomic sequencing. IAV genome fragments were recovered using probe capture-based sequencing from 74 sediment specimens that previously tested positive for IAV genomic material by RT-qPCR. (**A**) The length of each IAV genome fragment was estimated by FindFlu, a tool that aligned fragment sequences to a database of 555,364 IAV reference sequences (collected globally from avian, swine, and human hosts). Fragment length estimates were calculated from the start and end coordinates of these alignments. (**B**) FindFlu also estimated how much each fragment covered of its segment of origin by dividing the estimated fragment length by the length of the reference sequences to which it aligned.

FindFlu determines the segment and subtype of IAV fragments by aligning them to IAV reference sequences. It also uses these alignments to estimate the length of each recovered IAV fragment. For these specimens, the median IAV fragment length was 370 nucleotides, but lengths ranged from 104 to 2,113 nucleotides (Fig. 5A). FindFlu also uses these estimated fragment lengths to calculate how much each recovered fragment covers of its best-matching reference sequences (Fig. 5B). In this study, the median IAV fragment represented only 24% of the segment from which it originated, but some fragments represented up to 99% of their segment of origin.

### Assessing the presence of highly pathogenic goose/Guangdong/96 lineage H5 viruses

Next, we focused our analysis on H5 fragments due to the global importance of the highly pathogenic goose/Guangdong/96 (gs/Gd) lineage (21). Viruses in this H5 lineage have caused numerous outbreaks in poultry and humans since it emerged in the mid-1990s. When specimen collection for this study began, gs/Gd viruses had not been detected in North America since the end of a previous epizootic in 2015 (26), but they were an escalating problem across Eurasia (27–30). As such, local AIV surveillance priorities during the study period were detecting incursions of Eurasian H5 viruses, especially those belonging to gs/Gd lineages that had previously infected poultry or humans. We designed our custom One Health probe panel to ensure broadly inclusive coverage of gs/Gd lineages, and we confirmed extensive coverage of clades within this lineage bioinformatically (Table S2).

To further characterize the H5 fragments we recovered, they were queried against H5 reference sequences annotated with lineage/clade, collection location, and host species. Strong alignments were obtained between the fragments and their best-matching reference sequences (Fig. S2). The median alignment identity was 99.3%, and the median alignment coverage was 99.8%. This indicated that the H5 IAVs detected in these sediment specimens were very similar to previously described H5 IAVs, so their characteristics could be confidently inferred from the annotations of their best-matching reference sequences.

**Fig 6:**
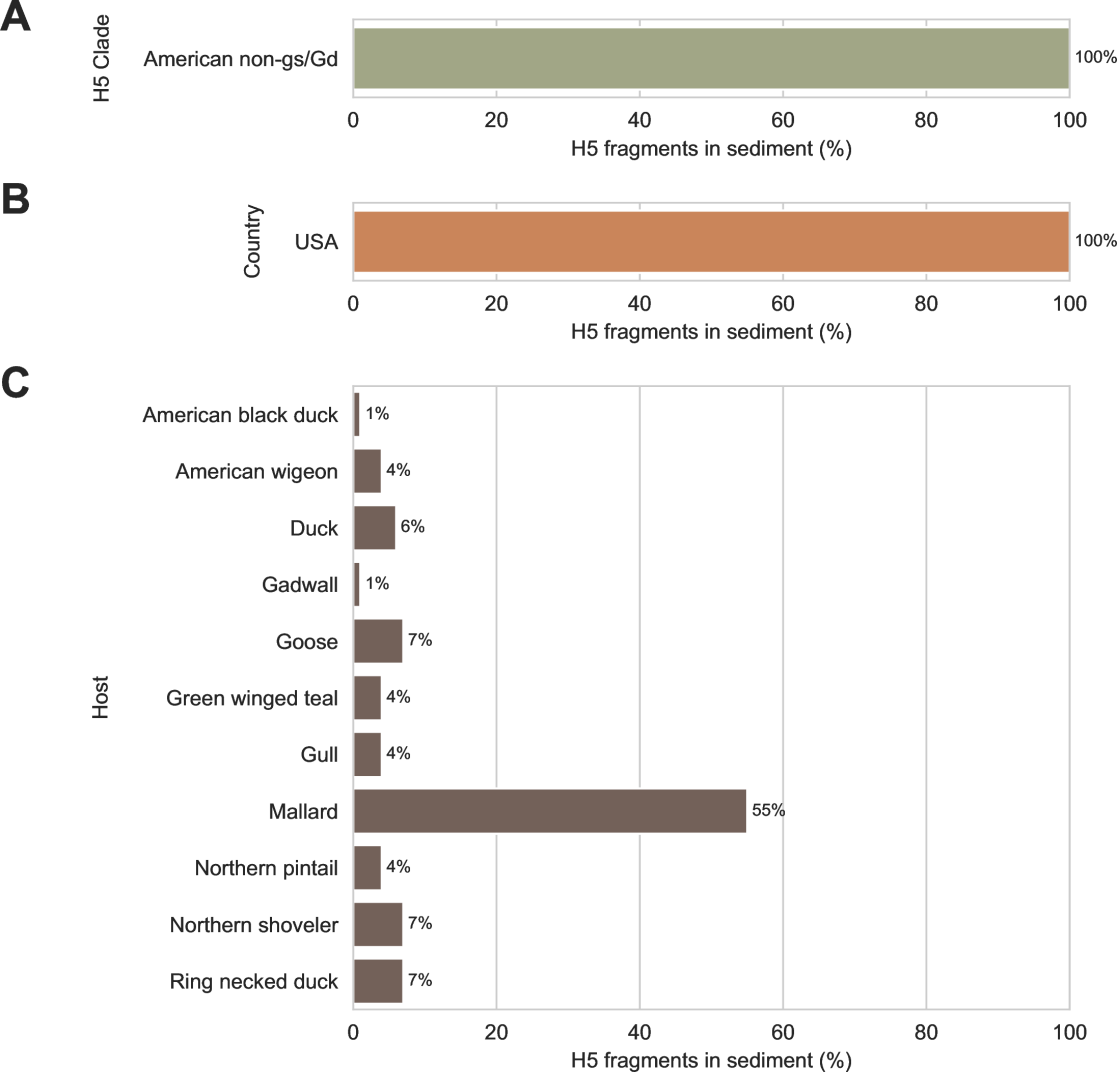
Lineage/clade, collection location, and host species of best-matching reference sequences for H5 genomic fragments detected in wetland sediment. Ninety-three fragments of H5 subtype HA genome segment were recovered using probe capture-based sequencing from 74 sediment specimens that previously tested positive for influenza A virus genomic material by RT-qPCR. Recovered H5 fragments were aligned against 6,041 H5 subtype HA segment reference sequences annotated with lineage/clade, collection location, and host species. Best matches were identified by alignment bitscores. (**A**) All H5 fragments had their best matches to reference sequences belonging to American non-goose/Guangdong (gs/Gd) lineages. (**B**) All H5 fragments had their best matches to reference sequences collected in the United States of America (USA). (**C**) All H5 fragments had their best matches to reference sequences collected from waterfowl and shorebird species.

None of the recovered H5 fragments had their best alignments to gs/Gd reference sequences; all best matches were to viruses belonging to American non-gs/Gd lineages (Fig. 6A). Furthermore, no incursions of Eurasian H5s were detected in this study; all recovered H5 fragments had their best alignments to viruses collected in North America (Fig. 6B). Finally, none of the recovered H5 fragments had their best alignments to IAVs that were collected from poultry or humans (Fig. 6C). All best alignments were to viruses collected from waterfowl and shorebirds.

**Fig 7:**
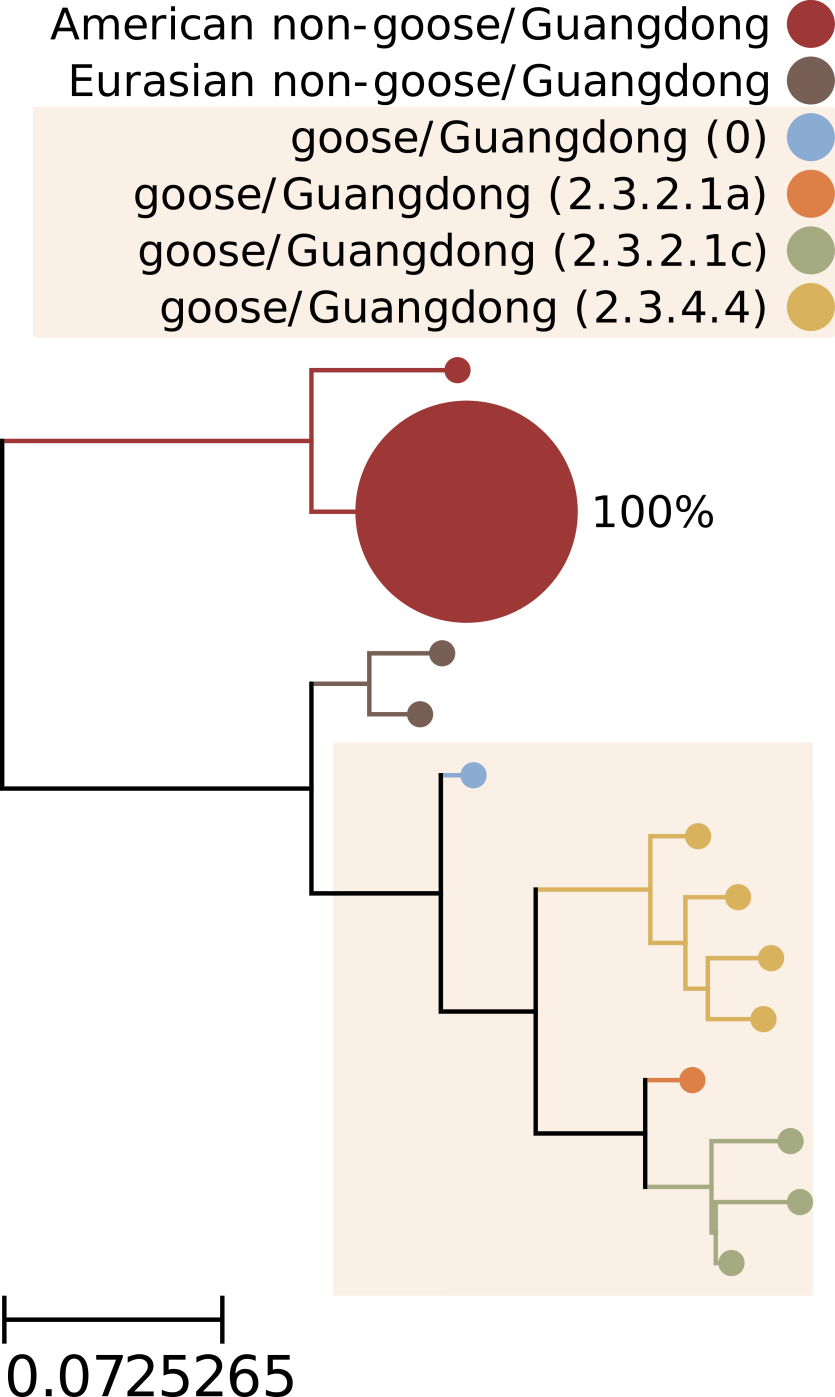
Phylogenetic context of H5 subtype influenza A viruses detected in wetland sediment by probe capture-based targeted genomic sequencing. A proxy phylogenetic tree was constructed from 147 recent HA segment nucleotide reference sequences belonging to the H5 subtype. Reference sequences were collected globally since 2018 (the past 5 years, inclusive). The HA segment sequence from the prototypical goose/Guangdong/96 lineage (GenBank accession NC_007362) was also included to represent clade 0 of this lineage. Monophyletic groups of highly similar sequences (all leaves within 0.025 substitutions/site of their common ancestor) were collapsed into single leaves for visual clarity. Leaves were colored according to their H5 lineage and clade. Background shading was applied to Gs/Gd lineage clades. Ninety-three fragments of H5 subtype HA segments were recovered from sediment specimens. These H5 fragments were aligned to the reference sequences composing the proxy tree. For each tree leaf, the percentage of recovered H5 fragments whose best-matching reference sequences belonged to the leaf was calculated. These percentages were indicated beside each leaf and used to scale leaf sizes.

We also evaluated the phylogenetic relationship of the H5 viruses in these specimens to the gs/Gd lineage. Direct phylogenetic comparison was complicated by the fragmentary and incomplete sequences recovered from the sediment. We deliberately did not attempt to assemble fragments into larger contigs; since there was evidence of multiple viruses in many of these specimens (Fig. 3C), we did not want to inadvertently create artificial chimeric H5 segment sequences that did not exist in the specimens. Instead, we constructed a proxy phylogenetic tree of H5 reference sequences; then, we aligned the H5 fragments that we recovered to these reference sequences to situate the fragments in their phylogenetic context (Fig. 7). This analysis indicated that the H5 IAVs that we detected were only distantly related to gs/Gd viruses, diverging from each other before the common ancestor of all gs/Gd lineage IAVs emerged in the mid-1990s.

Finally, we assessed the virulence of the H5 IAVs in the sediment by characterizing HA cleavage sites on recovered fragments. A common feature of highly pathogenic AIVs is the presence of polybasic amino acid insertions in this cleavage site (27, 31). We identified nine H5 fragments on which the HA cleavage site had been sequenced. All nine of these fragments contained the same canonical low-pathogenicity H5 cleavage site motif: PQRETRGLF.

## DISCUSSION

In this study, we demonstrated that our custom targeted genomic sequencing method can be used to effectively recover and characterize IAV genomic material in wetland sediment. All segments of the IAV genome were detected (Fig. 1), and diverse HA and NA subtypes were observed (Fig. 3). Multiple HA and NA subtypes were frequently detected in the same specimen (Fig. 3), highlighting this advantage of environmental surveillance. The diversity of subtypes that we observed showed that the custom probe panel designed for this study is broadly inclusive of diverse AIVs. It also revealed high HA and NA subtype richness among wild bird communities in the wetlands visited during the study period. It is important to note that the biases of environmental surveillance are not yet understood, so conclusions about the relative prevalence of IAV subtypes in the wild bird community should not be directly inferred from these sediment results.

This method succeeded in recovering IAV genome fragments from specimens with low abundance of viral genomic material (Fig. 2). Significant negative monotonic relationships were observed between screening RT-qPCR Ct values, the number of IAV genomic fragments recovered, and the number of IAV genome segments detected in a specimen (Fig. 2). The practical implication of these results is that specimens with lower screening RT-qPCR Ct values (i.e., higher abundance of viral genomic material) should be prioritized when sequencing capacity is limited.

This method’s ability to recover IAV genome fragments from specimens with low abundance of viral genomic materials means that detections of particular segments or subtypes might rely on the recovery of limited numbers of genome fragments. This study demonstrated that these detections are credible. Even when the number of fragments detected in a specimen were limited, those fragments were described by hundreds to thousands of read pairs (Fig. 4A). Furthermore, there was no evidence of cross-contamination in the six independent negative controls (Table S3), and chimeras and index hops were rare in data processed by HopDropper. The lack of evidence for false detections in this study reflects several method design choices that were made to increase confidence in results. IAV material is not used as positive control material so it cannot contaminate specimens. Negative controls contain sufficient background material to provide sensitive detection of low-level cross-contamination. UMIs are used during library construction to enable effective chimeric artefact removal.

This study also highlighted that the incomplete and fragmentary nature of IAV genomic material recovered from these specimens is a constraint of using wetland sediment for genomics-based AIV surveillance. Only 20% of specimens had fragments from all eight IAV genome segments recovered by probe capture-based targeted genomic sequencing (Fig. 1C). Most fragments were short and only represented small regions of the IAV genome segment from which they originated (Fig. 5).

Some longer fragments were recovered, but only up to 300 nucleotides were sequenced from each end of these fragments. This is an important trade-off for this method: Illumina short read platforms may leave the middles of longer fragments undescribed, but their high single-read accuracy and paired-end chemistry are instrumental for UMI-based single-fragment resolution and chimeric artefact removal. This trade-off seems prudent when enrichment and amplification are necessary to sequence fragmentary genomic material originating from multiple viruses in complex, challenging environmental specimens. If sequencing further along fragments is desired, paired-end sequencing runs could be performed with asymmetrical read lengths, e.g., 25 cycles for one read (to capture the UMI on that end of the molecule) followed by 575 cycles for the other read (to sequence further along the fragment). Alternatively, data generated by this method could be used to identify libraries containing long fragments of particularly high interest; these libraries could then be individually reflexed to a long-read platform.

In many applications, it is routine and appropriate to assemble fragmentary sequences into larger contigs. Full genomes might be instrumental for comparing genetic similarity between strains or constructing trees for phylodynamic analyses. The results from this study suggest that assembling the fragments recovered from sediment is not prudent. Genomic material from multiple viruses was often present in a single specimen (Fig. 3C). Thus, assembling fragments may combine sequences originating from different viruses and create fictitious genomes. Each distinct fragment should be analyzed independently, and these fragments must be the unit of analysis for surveillance.

Fortunately, the IAV genome fragments recovered from sediment provided useful information that addressed practical surveillance questions. Strong alignments to H5 reference sequences indicated that the H5 fragments in these specimens likely originated from American non-gs/Gd lineages that commonly circulate in North American resident waterfowl and shorebirds (Fig. 6). This was also supported by proxy phylogenetic analysis (Fig. 7). Based on the positions in this tree of (i) the prototypical gs/Gd sequence from 1996, (ii) contemporary gs/Gd clades, and (iii) the branch to which the recovered H5 fragments aligned, we surmised that the H5 viruses detected in these specimens were separated from gs/Gd viruses by several decades of evolution.

It is important to note the limitations of comparing recovered fragments to reference sequences, though. One must consider whether best matches rely on coincidental genetic similarity, especially when short fragment lengths could limit the robustness of alignments. One must also consider that IAV diversity in wild birds is undersampled, so a fragment’s best-matching reference sequences might be only distantly related to its true lineage if that lineage is absent from genomic databases. Finally, one must also consider biases in reference sequence databases. This is especially important when database contents are largely derived from surveillance programs and outbreak responses which have their own sampling biases and differ in scale between jurisdictions.

To mitigate these issues, comparisons to reference sequences should be limited to well-characterized subtypes with well-defined risk lineages, e.g., H5 viruses, and they should be restricted to alignments with high identity and query coverage. Conclusions drawn from these comparisons should also be limited to broad, qualitative observations about host range and geographic origin. For example, they could be used to distinguish Eurasian H5s from American H5s or to distinguish H5s that circulate solely in wild birds from those with a history of spillover into livestock and humans. Indeed, the absence of livestock and human viruses among a fragment’s best matches may have a high negative predictive value as these hosts are overrepresented in reference sequence databases and far less likely to be undersampled.

These limitations are not unique to probe capture-based sequencing or genomic material obtained from environmental specimens, however. All genomic surveillance programs must contend with these interpretation challenges when attempting to predict viral phenotype and spillover risk from best-matching reference sequences and phylogenetic context. That is why this study assessed pathogenicity more directly by interrogating recovered fragments for genetic markers of virulence, specifically H5 cleavage sites. We detected only canonical, monobasic low-pathogenicity cleavage motifs, corroborating the conclusions drawn above from the best-matching reference sequences and phylogenetic context of recovered H5 fragments. While we focused on HA cleavage sites, this same concept could be applied to other phenotypic markers of virulence and host range (32–35).

Taken together, these H5 results from sediment are consistent with a low prevalence of Eurasian-origin, high-pathogenicity gs/Gd lineage IAVs in BC during the study period. There were no IAV outbreaks in BC poultry during the study period; the ongoing clade 2.3.4.4b H5N1 epizootic did not arrive in BC until April 2022, 3 months after specimen collection for this study had ended. There were also no H5 viruses detected in BC birds by passive surveillance programs during the study period, although a single bald eagle fatality was detected shortly after the end of the study in February 2022 (36). A Eurasian gs/Gd H5 IAV was recovered from this bald eagle, but it was unrelated to the ongoing H5N1 epizootic, and it was not linked to any local poultry outbreaks (36).

Overall, this study demonstrated that environmental surveillance of wild bird habitats using targeted genomic sequencing could become a powerful, complementary approach to traditional bird-based strategies. At this point, it appears best suited for early detection of specific lineages associated with spillover risk in the local wild bird community. Further work will be needed to understand how it can serve other surveillance objectives, such as understanding transmission cycles, spatial and temporal dynamics, and prevalence of different IAV lineages in different wildlife hosts (13, 14). These questions will require well-designed and deliberate collection schemes to provide an ecologically representative sample of IAV biodiversity. Designing representative collection schemes remains a challenge, however, even for bird-based surveillance, so it was beyond the scope of this manuscript to attempt this for environmental surveillance using these data. Nonetheless, these results show that our targeted genomic sequencing method can be used to generate the data required for future studies attempting such an analysis.

The laboratory protocols and bioinformatic used in this study tools are flexible, and they could be adapted for other surveillance applications. They can accommodate RNA extracted from various types of specimens, not only wetland sediment. This expands their use to animal-based AIV surveillance as a culture-free method for direct sequencing of IAVs in bird swabs. This would avoid the extensive biocontainment infrastructure required for culturing suspected highly pathogenic AIVs, and it would be useful for sequencing swabs with low viral loads that fail conventional whole genome sequencing methods. Sequencing of wetland sediment and bird swabs with this method would be easily scaled and parallelized; sediment and swab specimens could be processed simultaneously on the same library construction plates, captured in the same reaction, and sequenced on the same run, thereby increasing throughput and decreasing cost per specimen.

The One Health design of our custom probe panel further expands the types of specimens that could be assayed to include clinical specimens from other animals (e.g., swine and humans) as well as diverse environmental specimens (e.g., material from swine barns and agricultural fairs, filtered air from building HVAC systems, and municipal wastewater) (37–41). Additionally, these specimens often contain other pathogens of importance to agriculture and public health, and probe panels could be expanded for simultaneous multi-pathogen detection (42–45). In this way, the probe capture-based targeted genomic sequencing method demonstrated here could provide a powerful general-purpose toolkit for pathogen surveillance.

## MATERIALS AND METHODS

### Specimen collection

Sediment specimens were collected from 22 wetlands across the Metro Vancouver and Lower Mainland region of British Columbia, Canada. Superficial sediment was collected from twenty separated sites at each wetland, providing a total of 440 specimens for the study. Specimen collection occurred between 6 October 2021 and 17 January 2022. All 20 sites in a wetland were sampled on the same day. Wetland locations were selected in consultation with local biologists to determine areas that were likely to have high abundance of wild waterfowl. Within a wetland, sampling locations were selected to maximize potential of use by waterfowl (e.g., evidence on shoreline of recent waterfowl usage) and ease of access to submerged sediment (e.g., water depth of less than 0.5 m) and to represent as much of the spatial extent of the wetland as possible. Sampling locations within a wetland were separated by 2 m or more. At each sampling location, biologists walked 1 to 2 m into the water and scooped the superficial layers of sediment into a 120-mL sterile urine collection container while wearing sterile nitrile gloves. Environmental data were then collected at each sampling location, including the geographic coordinates, an estimated water depth, water pH, water salinity, water temperature, and the presence or absence of fresh waterfowl feces at the shoreline.

Containers of sediment were brought back to the laboratory and kept at 4°C until pre-processing could occur. During pre-processing, excess water was decanted, and large chunks of organic debris (e.g., leaves, plant roots, and rocks) were removed. The remaining material was manually stirred with a sterile metal scoopula to homogenize it; then, 10 to 12 mL of the remaining material was placed into a 15-mL conical tube. The outsides of the tubes were wiped clean, disinfected with a 10% bleach solution, and then placed into a −80°C freezer until RNA extraction.

### Total RNA extraction from sediment specimens and RT-qPCR screening for IAV genomic material

Total RNA was extracted from 435 of 440 total sediment specimens collected for this study (five specimens had insufficient sediment for extraction). Total RNA was extracted from 2 g of sediment using the Qiagen RNeasy PowerSoil Total RNA kit (#12866). A chloroform extraction was added to the manufacturer’s protocol to remove additional PCR inhibitors. After the phenol:chloroform:isoamyl alcohol (pH 6.5–8.0) extraction step in the manufacturer’s protocol, the aqueous phase was transferred to a new container and then mixed with an equal volume of chloroform. Phases were separated by centrifugation; then, this chloroform extraction was repeated on the aqueous phase. The manufacturer’s protocol was resumed after the second chloroform extraction. RNA was eluted in 30 µL of nuclease-free water and stored at −80°C.

IAV genomic material was detected by RT-qPCR targeting the matrix (M) segment (Table 1) (46). Twenty-five-microliter reactions were prepared with Applied Biosystems AgPath-ID One-Step RT PCR reagents (#4387391), 400 nM each of primers M52C and M253R (Table 1), 120 nM of the FAM-labeled probe M96C (Table 1), 3 µg of New England BioLabs T4 Gene 32 Protein (#M0300), and 2 µL of RNA extracted from sediment specimens. Reactions were incubated with the following cycling conditions: 1 cycle of 45°C for 10 min, 1 cycle of 95°C for 10 min, 45 cycles of 95°C for 15 s followed by 60°C for 60 s. Reactions were run on an Applied Biosystems 7500 Fast Real-Time PCR System using a fixed critical threshold of 0.05 for all reactions. Following common clinical practice, a Ct value of 40 was selected as the cut-off for specimen positivity. Screening RT-qPCRs was allowed to proceed for an additional five cycles, however, to identify suspect-positive specimens and assess their value for surveillance. Specimens were called suspect positive if they had Ct values greater than 40 or if their amplification curves trended toward the critical threshold in the final PCR cycle.

**TABLE 1:** Sequences of oligonucleotides used in this study

Oligo name	Oligo purpose	Oligo sequence (5′ to 3′)	Reference
M52C	Forward primer for IAV M segment detection	CTTCTAACCGAGGTCGAAACG	Fouchier et al. (46)
M253R	Reverse primer for IAV M segment detection	AGGGCATTTTGGACAAAKCGTCTA	Fouchier et al. (46)
M96C	Taqman probe for IAV M segment detection	CCGTCAGGCCCCCTCAAAGCCGA	Fouchier et al. (46)
control_oligo	Positive control target for probe capture-based targeted genomic sequencing	GTTCTTAGCTATTGCGCTTCCGCA ATNNNBANNNDCNNNHGGCCAAT ACAGTTGGAGAGCGTGTTGGCGAA TATAAGCCACTCGCGAATGGTCCG CCAGGCTAGCTTCATTCGTCGATG CACCGTATATGGTCATCTATATAT CTAACTCGACACAACACHNNNGD NNNTBNNNATTGCGTGATACAGCA AGAGACAACG	This study
control_oligo_f	Forward primer for amplifying and detecting control oligo	CGTTGTCTCTTGCTGTATCACGC	This study
control_oligo_r	Reverse primer for amplifying and detecting control oligo	GTTCTTAGCTATTGCGCTTCCGC	This study
control_oligo_p	Taqman probe for detecting control oligo	TGAAGCTAGCCTGGCGGACC	This study

### cDNA synthesis and library construction

Double-stranded cDNA was prepared from 11 µL of undiluted RNA using the Invitrogen SuperScript IV First-Strand Synthesis System (#18091200) and the Invitrogen Second Strand cDNA Synthesis Kit (#A48571). First- and second-strand syntheses were both performed according to the manufacturer’s protocols and then purified using 1.8× Agencourt AMPure XP-PCR Purification Beads (#A63881). Sequencing libraries were prepared from the total volume of purified cDNA using the Integrated DNA Technologies xGen cfDNA & FFPE DNA Library Preparation Kit (#10006202) according to the manufacturer’s protocol. Libraries were barcoded using the xGen UDI Primers Plate 1 (#10005922) with 15 cycles of PCR. Following barcoding PCRs, libraries were purified with 1.3× Agencourt AMPure XP-PCR Purification Beads (#A63881) and then eluted in 30 µL of nuclease-free water.

Libraries were prepared in batches of 15 sediment specimens and one batch control specimen. Sediment specimens were randomly assigned to six batches. All specimens in the same batch were prepared on the same reaction plates and from the same reagent master mixes. Batch controls were composed of 500 ng of Invitrogen Universal Human Reference RNA (#QS0639) spiked with 40,000 copies of double-stranded control oligo. The sequence of the control oligo was generated randomly (Table 1); then, it was synthesized as an ssDNA Ultramer DNA Oligo by Integrated DNA Technologies (Coralville, Iowa, USA). Single-stranded control oligo was amplified by PCR as follows. Fifty-microliter reactions were prepared with New England BioLabs NEBNext Ultra II Q5 Master Mix (#M0544), 1 µM of each control oligo amplification primer (Table 1), and 20 million copies of single-stranded control oligo Ultramer as template. Reactions were incubated with the following cycling conditions: 1 cycle of 98°C for 30 s, 10 cycles of 98°C for 15 s followed by 65°C for 75 s, and 1 cycle of 65°C for 10 min. After amplification, double-stranded control oligo PCR products were purified using 1.2× Agencourt AMPure XP-PCR Purification Beads (#A63881) and then eluted in 25 µL of nuclease-free water.

To spike batch controls with the specified copies of double-stranded control oligo, the molarity of the purified double-stranded control oligo PCR product was determined by qPCR. Twenty-microliter reactions were prepared with New England BioLabs Luna Universal Probe qPCR Master Mix (#M3004), 250 nM of each control oligo amplification primer (Table 1), 250 nM of FAM-labeled control oligo detection probe (Table 1), and 2 µL of purified double-stranded control oligo PCR product. Reactions were run on an Applied Biosystems 7500 Fast Real-Time PCR System with the following cycling conditions: 1 cycle of 95°C for 60 s and 40 cycles of 95°C for 15 s followed by 60°C for 45 s. A dilution series of the single-stranded control oligo Ultramer stock was used as a standard curve for quantification.

Enrichment of control oligos in batch control specimens functioned as a positive control for library construction and probe capture. Absence of control oligos in sediment specimens following index hop removal (described below) functioned as a negative control for reagent contamination and cross-contamination between specimens. Absence of IAV fragments in batch control specimens also functioned as a negative control in the same way.

### One Health IAV probe panel design

IAV genome segment sequences were downloaded from the Influenza Research Database (www.fludb.org) (47) on 9 December 2021. Sequences were limited to those marked as complete from avian, swine, and human hosts. In total, 531,526 IAV genome segment nucleotide sequences were obtained. Separate sub-panels were designed for each IAV genome segment as follows. First, all reference sequences representing a segment were clustered at 99% nucleotide identity using VSEARCH cluster_fast (v2.21.0) without masking (-qmask none) (48). Cluster centroids were used as the input design space for ProbeTools *makeprobes* (v0.1.9) using batch sizes of 10 probes (-b 10), probe length of 120 nucleotides (-k 120), and a coverage endpoint of 95% (-c 95) (42). Sub-panels for each IAV genome segment were combined to create the final panel. Ten additional probes with randomly generated sequences were added for capturing synthetic spike-in control oligos, although only one of these synthetic controls was used in this study (described above). The final panel contained 9,380 probes (sequences provided in Supplemental Material 1). ProbeTools *capture* and *stats* (v0.1.9) were used to confirm extensive coverage by the final panel of reference sequences in the design space (Tables S1 and S2). The final panel was synthesized by Twist Biosciences (San Francisco, California, USA) with 0.02 fmol of each probe per reaction.

### Library pooling, hybridization probe capture, and genomic sequencing

dsDNA concentration was measured for each library using the Invitrogen Qubit dsDNA Broad Range kit (#Q32851) on the Invitrogen Qubit 4 Fluorometer. Three hundred nanograms of each library were pooled together; then, two independent capture replicates were performed on aliquots of the pool. For each capture replicate, two aliquots of 4 µg of the pool were captured separately. After this first capture, they were combined and subjected to an additional capture for further enrichment of IAV genomic material. This means that 8 µg of library pool was enriched for each independent capture replicate and 16 µg of library pool was enriched in total for the whole study.

Pooled library material was completely evaporated in a vacuum oven at 50°C and −20 mm Hg; then, hybridization reactions were prepared with our custom One Health IAV panel (described above), Twist Universal Blockers (#100578), and Twist Hybridization Reagents (#104178) according to the manufacturer’s protocol. Hybridization reactions were incubated at 70°C for 16 hours then washed with Twist Wash Buffers (#104178). Washing was performed according to the manufacturer’s protocol except the streptavidin bead slurry was resuspended in 22.5 µL of nuclease-free water instead of 45 µL prior to post-capture PCR. Post-capture PCR was performed on the total volume of bead slurry using Twist Equinox Library Amp Mix (#104178). Reactions were prepared and incubated according to the manufacturer’s protocol with 15 cycles of amplification. Following post-capture PCR, reactions were purified with the included DNA Purification Beads according to the manufacturer’s protocol. Purified captured library pools were eluted in 30 µL of nuclease-free water.

Molarity of double-captured library pools was determined using the New England BioLabs NEBNext Library Quant Kit for Illumina (#E7630). Double-captured library pools were also run on the Agilent TapeStation 2200 device using Agilent D1000 ScreenTape (#5067-5582) and D1000 reagents (#5067-5583) to obtain the peak fragment size, which was used to adjust molarity. Fifteen picomoles of double-captured library pool were sequenced on an Illumina MiSeq instrument using MiSeq v3 600 cycle reagent kits (#MS-102-3003) to generate 2 × 300 cycle paired-end reads. Each independent capture replicate was sequenced on its own run. The following adapter sequences were provided in the MiSeq sample sheet for on-instrument trimming: AGATCGGAAGAGCACACGTCTGAACTCCAGTCA and AGATCGGAAGAGCGTCGTGTAGGGAAAGAGTGT.

### Chimera and index hop removal and generation of consensus sequences for distinct fragments

Each MiSeq run was separately analyzed with HopDropper (v1.0.0) (https://github.com/KevinKuchinski/HopDropper). All FASTQ files generated in the run were analyzed, including sediment specimen libraries, control specimen libraries, and undetermined libraries. Fourteen-nucleotide intrinsic UMIs and 8-nucleotide extrinsic UMIs were assigned to each read, and extrinsic UMIs were limited to the 32 indices provided with the Integrated DNA Technologies xGen cfDNA & FFPE DNA Library Preparation Kit (#10006202). Fragments and their read pairs were only outputted if their UMI pair was observed at least 30 times. Fragment end consensus sequences were generated by sub-sampling up to 200 read pairs from each fragment. HopDropper defaults were used for other parameters.

### Identification and characterization of influenza A virus genome fragments

Fragment end consensus sequences generated by HopDropper were analyzed by FindFlu (v0.0.8) (https://github.com/KevinKuchinski/FindFlu). The FindFlu database used for this study was comprised of all complete segment nucleotide sequences in the Influenza Research Database (www.fludb.org) from avian, swine, and human hosts on 11 October 2022. IAV reference sequence was further filtered by length as follows: between 2,260 and 2,360 nucleotides for PB2 and PB1 segment sequences, between 2,120 and 2,250 nucleotides for PA segment sequences, between 1,650 and 1,800 nucleotides for HA segment sequences, between 1,480 and 1,580 nucleotides for NP segment sequences, between 1,250 and 1,560 nucleotides for NA segment sequences, between 975 and 1,030 nucleotides for M segment sequences, and between 815 and 900 nucleotides for NS segment sequences. The final database contained 169,098 avian-origin sequences, 70,918 swine-origin sequences, and 315,348 human-origin sequences (555,364 total sequences). IAV fragments from both probe capture replicates were combined for analyses in this study, except for analyses where probe capture replicates were explicitly considered separately. All fragment counts were based on UMI pair to ensure that IAV fragments were not double counted if they were enriched in both probe capture replicates.

The FindFlu fragment report provided the following for each IAV fragment: segment, subtype, number of copies sequenced, fragment length, segment coverage, alignment identity, and alignment query coverage. The FindFlu host, country, and H5 clade reports were used to calculate the percentage of IAV fragments having their best matches to reference sequences from various host species, collection countries, and H5 clades. In cases where IAV fragments had multiple best-matching reference sequences with multiple host/country/H5 clade annotations, each different host/country/H5 clade value observed was proportionally allocated a fraction of a fragment (1/*n* where *n* was the number of best-matching reference sequences the fragment had).

### Phylogenetic analysis of H5 fragments

Recent H5 segment reference sequences were downloaded from the Influenza Research Database (www.fludb.org) (47). All available complete H5 segment nucleotide sequences collected from 2018 onwards were downloaded on 6 November 2022. The prototypical goose/Guangdong/96 lineage HA sequence (GenBank accession NC_007362) was also included to represent clade 0. A multiple-sequence alignment was performed on the resulting collection of 147 H5 reference sequences using CLUSTAL W (v2.1) (49). A maximum-likelihood phylogenetic tree was constructed from the multiple sequence alignment and bootstrapped 100 times using PHYML (v3.3.20211231) (50). The resulting tree was analyzed and visualized with the ETE3 package (v3.1.2) in Python (v3.9.12) (51). Outlying branches were trimmed if their length exceeded three standard deviations of the mean branch length. For visual clarity, monophyletic groups of similar leaves were collapsed into a single leaf if all leaves were less than 0.025 substitutions/site from their common ancestral node. The length of the replacement leaf’s branch was set to the mean branch length of the collapsed leaves.

The best-matching reference sequences for each H5 fragment were determined as follows. The H5 fragment end consensus sequences generated by HopDropper were aligned to the H5 reference sequences composing the tree using blastn (v2.13.0) (52). A combined bitscore was generated for each fragment-reference sequence combination by adding together the bitscores from both fragment end consensus sequence alignments against that reference sequence. Each fragment’s best-matching reference sequences were those with which it had its maximum combined bitscore.

The percentage of H5 fragments having their best match to each reference sequence composing the tree was calculated as follows. The number of H5 fragments having their best match to a reference sequence was divided by the total number of H5 fragments then multiplied by 100. In cases where an H5 fragment had multiple best matches, that fragment was counted as 1/*n* of a fragment for each of their best matches, where *n* was that fragment’s number of best matches. When similar tree leaves were collapsed into a single leaf for visual clarity, the replacement leaf’s percentage of H5 fragments having their best match to it was determined by summing the percentages of its constituent leaves.

### H5 cleavage site characterization

H5 fragment end consensus sequences generated by HopDropper were translated and aligned to the prototypical goose/Guangdong/96 lineage HA amino acid sequence (GenBank accession NC_007362) using blastx (v2.13.0) (52). Only the best alignments (by bitscore) were retained for each fragment end consensus sequence. The position of each fragment end consensus sequence in the goose/Guangdong H5 amino acid sequence was determined from the alignment subject start and subject end coordinates. Fragment end consensus sequences containing the HA cleavage site were identified by finding fragments that spanned the coordinates 336 and 348. HA cleavage site motifs were then extracted from the aligned, translated sequences.

## Data Availability

Source codes for HopDropper and FindFlu are available at https://github.com/KevinKuchinski/. Raw sequencing reads generated during this study are available from the NCBI Short Read Archive as part of BioProject PRJNA926989. Influenza A virus genome fragments recovered in this study (following HopDropper and FindFlu analysis, as described above) have been included as a supplemental FASTA file (Supplemental Material 2).

## SUPPLEMENTAL MATERIAL

Supplemental material 1 aem.00842-23-s0001.txt One Health influenza A virus probe panel.

Supplemental material 2 aem.00842-23-s0002.txt Influenza A virus genome fragments recovered from sediment specimens.

Figure S1 aem.00842-23-s0003.pdf Recovered influenza A genome fragments stratified by specimen and segment/subtype.

Figure S2 aem.00842-23-s0004.pdf Alignment identity and coverage between recovered H5 genome fragments and their best-matching reference sequences.

Table S1 aem.00842-23-s0005.pdf *In silico* coverage of influenza A virus reference sequences by custom probe panel.

Table S2 aem.00842-23-s0006.pdf *In silico* coverage of H5 reference sequences by custom probe panel.

Table S3 aem.00842-23-s0007.pdf Probe capture and sequencing metrics.

ASM does not own the copyrights to Supplemental Material that may be linked to, or accessed through, an article. The authors have granted ASM a non-exclusive, world-wide license to publish the Supplemental Material files. Please contact the corresponding author directly for reuse.
