# Supplementary material for: Targeted genomic sequencing of avian influenza viruses in wetland sediment from wild bird habitats
Source: Appl Environ Microbiol. 2024 Jan 23;90(2):e00842-23. doi: 10.1128/aem.00842-23 (PMC10880596; doi:10.1128/aem.00842-23)
Supplement: Figure S1 — Recovered influenza A genome fragments stratified by specimen and segment/subtype. [file aem.00842-23-s0003.pdf]

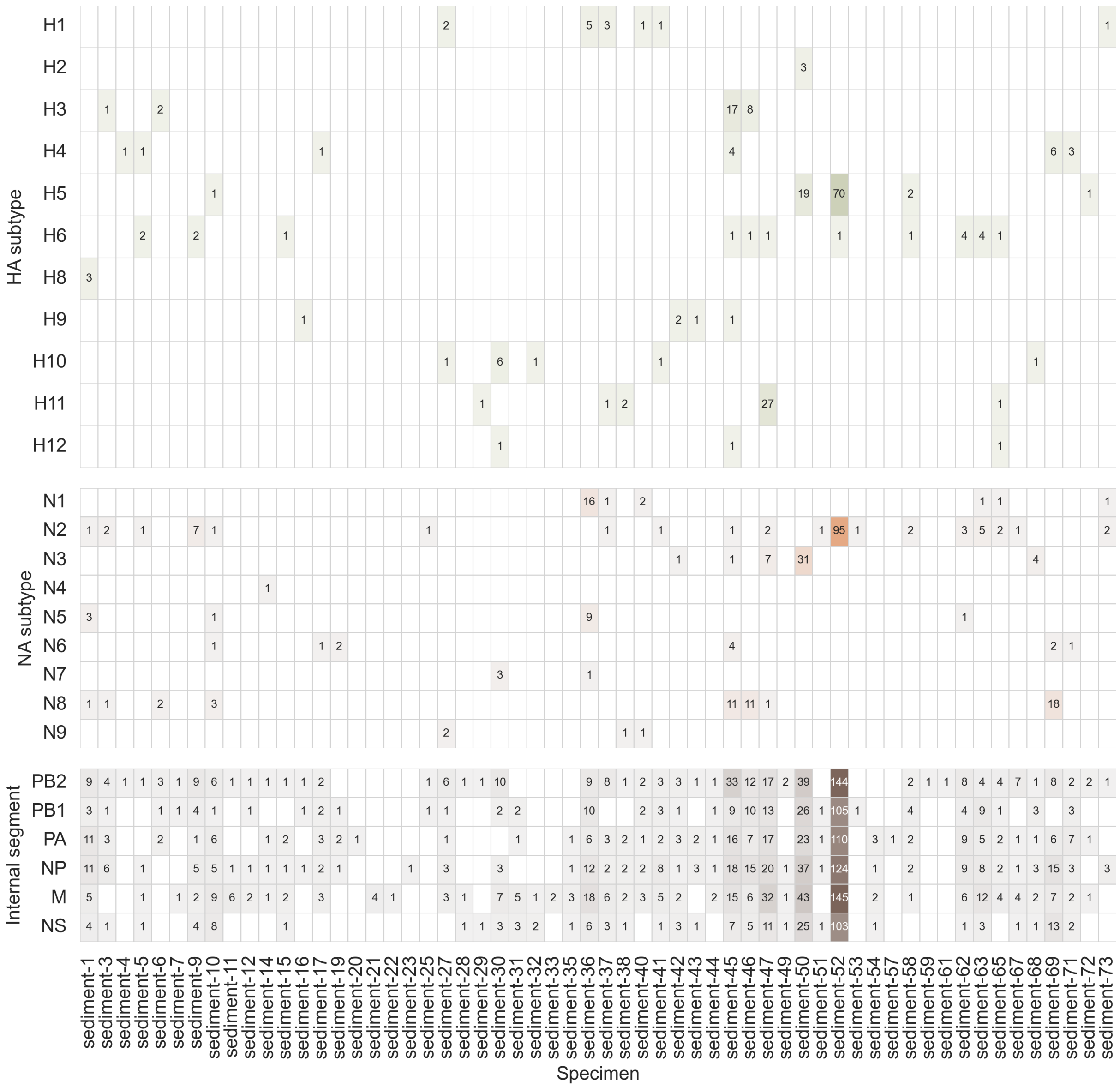

Figure S1: Number of influenza A virus genome fragments detected in wetlands sediment by probe capture-based targeted genomic sequencing. 2,312 fragments of influenza A virus (IAV) genome were recovered using probe capture-based sequencing from 74 sediment specimens that had previously tested positive for IAV genomic material by RT-qPCR. Numbers inside cells indicate the number of IAV fragments originating from a particular segment/subtype that were recovered from the corresponding specimen. Only specimens that contained IAV fragments were plotted.
