# Supplementary material for: Targeted genomic sequencing of avian influenza viruses in wetland sediment from wild bird habitats
Source: Appl Environ Microbiol. 2024 Jan 23;90(2):e00842-23. doi: 10.1128/aem.00842-23 (PMC10880596; doi:10.1128/aem.00842-23)
Supplement: Figure S2 — Alignment identity and coverage between recovered H5 genome fragments and their best-matching reference sequences. [file aem.00842-23-s0004.pdf]

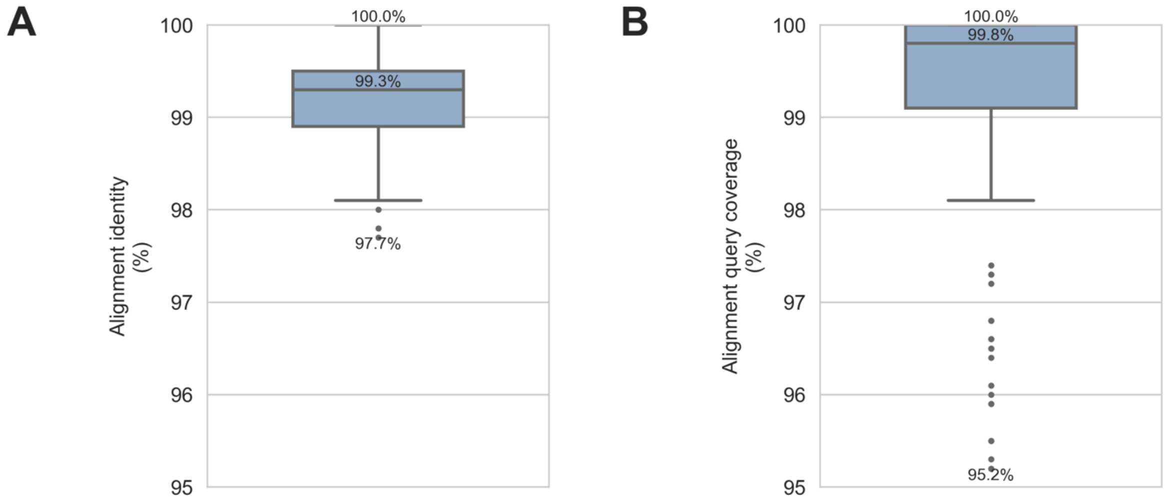

Figure S2: Haemagglutinin genome segment fragments originating from H5 subtype viruses were highly similar to H5 reference sequences. Recovered H5 fragments were aligned against 6,041 H5 subtype HA segment reference sequences annotated with lineage/clade, collection location, and host species. Best matches were identified by alignment bitscores. **A)** Nucleotide sequence identities were calculated by dividing the number of identical bases by the alignment length. **B)** Query sequence coverage values were calculated by dividing alignment lengths by query sequence lengths. The minimum, median, and maximum values in both distributions are indicated on the plots.
