# Supplementary material for: Targeted genomic sequencing of avian influenza viruses in wetland sediment from wild bird habitats
Source: Appl Environ Microbiol. 2024 Jan 23;90(2):e00842-23. doi: 10.1128/aem.00842-23 (PMC10880596; doi:10.1128/aem.00842-23)
Supplement: Table S1 — In silico coverage of influenza A virus reference sequences by custom probe panel. [file aem.00842-23-s0005.pdf]

Table S1: Custom probe panel provides broadly inclusive coverage of influenza A virus reference sequences. The ProbeTools capture and stats modules were used to predict *in silico* how well this study's custom panel of 9,380 probes covered 531,526 influenza A virus (IAV) reference sequences (collected globally from avian, swine, and human hosts). For each reference sequence, probe coverage was calculated as the number of nucleotide positions covered by at least one probe in the panel. The minimum, 5th percentile, median, and maximum probe coverage values were reported for each segment, subtype, and host category.

Segment	Subtype	Host	Reference sequences (#)	Minimum coverage (%)	Fifth percentile of coverage (%)	Median coverage (%)	Maximum coverage (%)
PB2	n/a	avian	19109	71.4	94.5	99.3	100.0
PB2	n/a	human	34153	86.4	91.8	97.8	100.0
PB2	n/a	swine	6767	73.0	95.6	99.1	100.0
PB1	n/a	avian	18173	70.9	94.2	99.7	100.0
PB1	n/a	human	28158	82.9	95.8	99.8	100.0
PB1	n/a	swine	5939	78.7	94.6	99.7	100.0
PA	n/a	avian	19990	62.6	93.9	99.9	100.0
PA	n/a	human	34391	80.7	93.9	98.1	100.0
PA	n/a	swine	6919	78.0	94.9	99.7	100.0
HA	all	avian	26173	62.1	93.9	99.9	100.0
HA	all	human	49011	82.3	98.5	99.8	100.0
HA	all	swine	11342	67.9	95	99.9	100.0
HA	H1	avian	916	73.3	91.6	98.1	100.0
HA	H1	human	21844	82.3	98.5	100.0	100.0
HA	H1	swine	7899	72.2	95.1	99.8	100.0
HA	H2	avian	530	81.7	90	99.4	100.0
HA	H2	human	95	93.6	98.8	99.6	100.0
HA	H2	swine	2	97.5	97.6	98.1	98.7
HA	H3	avian	2269	66.6	92.4	99.1	100.0
HA	H3	human	26651	89.6	98.5	99.0	100.0
HA	H3	swine	3374	67.9	94.7	99.9	100.0
HA	H4	avian	1928	72.8	93.9	99.4	100.0
HA	H4	human	1	100.0	100	100.0	100.0
HA	H4	swine	5	97.3	97.3	99.4	100.0
HA	H5	avian	5555	75.4	96.7	100.0	100.0
HA	H5	human	246	94.2	98.5	100.0	100.0
HA	H5	swine	33	98.8	99.4	100.0	100.0
HA	H6	avian	1902	77.4	94.2	99.8	100.0
HA	H6	swine	2	96.6	96.8	98.3	100.0
HA	H7	avian	2367	70.6	93.8	100.0	100.0
HA	H7	human	152	93.2	96.1	100.0	100.0
HA	H7	swine	3	85.7	86.9	98.0	98.0
HA	H8	avian	160	79.6	92.8	98.7	100.0
HA	H9	avian	7364	73.1	96.2	100.0	100.0
HA	H9	human	18	87.9	93.3	100.0	100.0
HA	H9	swine	22	87.5	92.1	97.6	100.0
HA	H10	avian	1231	81.8	92.8	99.9	100.0
HA	H10	human	4	98.5	98.5	98.5	98.5
HA	H10	swine	1	99.4	99.4	99.4	99.4
HA	H11	avian	736	77.5	93.3	99.9	100.0
HA	H11	swine	1	82.7	82.7	82.7	82.7
HA	H12	avian	344	78.5	92.6	98.1	100.0
HA	H13	avian	546	75.4	91	97.0	100.0
HA	H14	avian	39	79.3	85	100.0	100.0

Segment	Subtype	Host	Reference sequences (#)	Minimum coverage (%)	Fifth percentile of coverage (%)	Median coverage (%)	Maximum coverage (%)
HA	H15	avian	20	62.1	62.1	79.4	100.0
HA	H16	avian	266	69.2	81.0	98.4	100.0
NP	n/a	avian	19897	67.9	94.7	99.5	100.0
NP	n/a	human	36140	71.8	93.7	96.7	100.0
NP	n/a	swine	7168	68.8	94.2	98.3	100.0
NA	all	avian	20401	63.5	93.2	99.7	100.0
NA	all	human	43304	74.7	98.6	100.0	100.0
NA	all	swine	12126	69.4	95.8	100.0	100.0
NA	N1	avian	4446	78.8	95.0	98.8	100.0
NA	N1	human	19974	74.7	98.5	99.9	100.0
NA	N1	swine	4936	76.7	96.0	99.6	100.0
NA	N2	avian	5341	79.9	94.7	99.8	100.0
NA	N2	human	23200	86.9	99.7	100.0	100.0
NA	N2	swine	7171	69.4	95.8	100.0	100.0
NA	N3	avian	1681	74.8	91.7	98.5	100.0
NA	N3	human	2	95.7	95.8	96.4	97.0
NA	N3	swine	4	98.1	98.1	99.0	99.9
NA	N4	avian	378	81.1	90.0	99.8	100.0
NA	N4	human	1	96.3	96.3	96.3	96.3
NA	N5	avian	567	77.7	89.7	99.2	100.0
NA	N5	swine	1	94.3	94.3	94.3	94.3
NA	N6	avian	2930	78.3	92.9	99.9	100.0
NA	N6	human	4	100.0	100.0	100.0	100.0
NA	N6	swine	8	93.6	93.7	99.9	100.0
NA	N7	avian	1078	63.5	94.0	100.0	100.0
NA	N7	human	4	97.5	97.5	98.2	98.8
NA	N7	swine	1	74.1	74.1	74.1	74.1
NA	N8	avian	2528	74.9	92.7	99.8	100.0
NA	N8	human	5	99.8	99.8	100.0	100.0
NA	N8	swine	3	89.2	89.2	89.2	97.0
NA	N9	avian	1452	72.9	93.3	100.0	100.0
NA	N9	human	114	95.5	98.1	100.0	100.0
NA	N9	swine	2	100.0	100.0	100.0	100.0
M	n/a	avian	20263	77.0	92.7	100.0	100.0
M	n/a	human	40456	78.2	89.5	99.8	100.0
M	n/a	swine	9860	73.7	98.2	100.0	100.0
NS	n/a	avian	20810	66.5	95.4	99.8	100.0
NS	n/a	human	34027	76.1	98.3	100.0	100.0
NS	n/a	swine	6949	64.4	96.2	100.0	100.0
