# Supplementary material for: Targeted genomic sequencing of avian influenza viruses in wetland sediment from wild bird habitats
Source: Appl Environ Microbiol. 2024 Jan 23;90(2):e00842-23. doi: 10.1128/aem.00842-23 (PMC10880596; doi:10.1128/aem.00842-23)
Supplement: Table S2 — In silico coverage of H5 reference sequences by custom probe panel. [file aem.00842-23-s0006.pdf]

Table S2: Custom probe panel provides broadly inclusive coverage of H5 subtype haemagglutinin segment reference sequences from diverse clades of the goose/Guangdong/96 lineage. The ProbeTools captures and stats modules were used to predict *in silico* how well this study's custom panel of 9,380 probes covered 5,834 H5 subtype haemagglutinin (HA) segment reference sequences (collected globally from avian, swine, and human hosts). For each reference sequence, probe coverage was calculated as the number of nucleotide positions covered by at least one probe in the panel. The minimum, 5th percentile, median, and maximum probe coverage values were reported for each segment, subtype, and host category.

H5 clade	Host	Reference sequences (#)	Minimum coverage (%)	Fifth percentile of coverage (%)	Median coverage (%)	Maximum coverage (%)
0	avian	61	94.7	99.6	100.0	100.0
0	human	6	100.0	100.0	100.0	100.0
0	swine	1	100.0	100.0	100.0	100.0
1	avian	219	98.0	98.6	100.0	100.0
1	human	49	98.5	98.5	99.9	100.0
1,2,3,5,6,8,9-like	avian	2	96.5	96.7	98.2	100.0
1,2,5,6,8,9-like	avian	4	100.0	100.0	100.0	100.0
1,2,8-like	avian	3	99.6	99.6	100.0	100.0
1.1	avian	29	99.6	99.6	100.0	100.0
1.1.1	avian	9	95.7	96.2	99.9	100.0
1.1.1	human	3	95.3	95.5	97.0	100.0
1.1.2	avian	89	90.7	94.3	99.9	100.0
1.1.2	human	25	94.2	95.7	100.0	100.0
2-like	avian	4	99.8	99.8	100.0	100.0
2.1-like	avian	1	100.0	100.0	100.0	100.0
2.1.1	avian	23	95.5	99.3	100.0	100.0
2.1.1	swine	4	100.0	100.0	100.0	100.0
2.1.2	avian	9	99.8	99.8	100.0	100.0
2.1.2	human	6	99.4	99.4	99.6	99.6
2.1.3	avian	28	99.8	99.8	100.0	100.0
2.1.3	swine	5	99.8	99.8	100.0	100.0
2.1.3.1	avian	16	95.5	95.5	100.0	100.0
2.1.3.2	avian	134	94.7	95.1	100.0	100.0
2.1.3.2	human	33	95.2	95.7	100.0	100.0
2.1.3.2a	avian	21	99.2	99.3	100.0	100.0
2.1.3.2b	avian	4	99.3	99.3	99.8	100.0
2.1.3.3	avian	10	100.0	100.0	100.0	100.0
2.1.3.3	swine	4	100.0	100.0	100.0	100.0
2.2	avian	330	94.4	99.6	100.0	100.0
2.2	human	1	100.0	100.0	100.0	100.0
2.2-like	avian	15	99.8	99.8	99.9	100.0
2.2.1	avian	227	99.2	99.8	99.9	100.0
2.2.1	human	50	95.4	99.8	99.9	100.0
2.2.1.1	avian	76	95.5	99.4	100.0	100.0
2.2.1.1a	avian	58	95.4	96.6	99.9	100.0
2.2.1.2	avian	279	93.0	99.4	99.8	100.0
2.2.1.2	human	8	99.8	99.8	99.8	100.0
2.2.2	avian	31	99.3	99.6	100.0	100.0
2.2.2-like	avian	1	100.0	100.0	100.0	100.0
2.2.2.1	avian	22	99.5	99.8	99.8	99.8
2.2.2.1	human	2	99.8	99.8	99.8	99.8
2.3-like	avian	2	100.0	100.0	100.0	100.0
2.3.1	avian	2	100.0	100.0	100.0	100.0
2.3.2	avian	19	99.6	99.9	100.0	100.0

H5 clade	Host	Reference sequences (#)	Minimum coverage (%)	Fifth percentile of coverage (%)	Median coverage (%)	Maximum coverage (%)
2.3.2.1	avian	45	98.9	99.6	100.0	100.0
2.3.2.1	human	1	100.0	100.0	100.0	100.0
2.3.2.1-like	avian	1	100.0	100.0	100.0	100.0
2.3.2.1a	avian	328	91.5	96.6	99.9	100.0
2.3.2.1a	human	2	98.9	99.0	99.4	99.9
2.3.2.1b	avian	31	95.7	99.1	100.0	100.0
2.3.2.1b	swine	1	100.0	100.0	100.0	100.0
2.3.2.1c	avian	802	92.5	97.6	100.0	100.0
2.3.2.1c	human	1	100.0	100.0	100.0	100.0
2.3.2.1c	swine	1	100.0	100.0	100.0	100.0
2.3.3,4-like	avian	1	100.0	100.0	100.0	100.0
2.3.4	avian	142	94.2	99.4	100.0	100.0
2.3.4	human	31	99.5	99.9	100.0	100.0
2.3.4	swine	1	99.9	99.9	99.9	99.9
2.3.4-like	avian	1	100.0	100.0	100.0	100.0
2.3.4.1	avian	26	95.0	99.6	100.0	100.0
2.3.4.1	human	6	99.4	99.4	100.0	100.0
2.3.4.2	avian	26	94.2	99.7	100.0	100.0
2.3.4.2	human	3	99.7	99.7	100.0	100.0
2.3.4.3	avian	50	99.6	99.6	100.0	100.0
2.3.4.3	human	13	96.9	98.5	100.0	100.0
2.3.4.4	avian	1069	88.3	97.8	99.8	100.0
2.3.4.4	human	4	99.9	99.9	100.0	100.0
2.3.4.4	swine	2	99.9	99.9	99.9	99.9
2.4	avian	17	100.0	100.0	100.0	100.0
2.5	avian	13	100.0	100.0	100.0	100.0
3	avian	17	99.9	99.9	100.0	100.0
3	human	1	100.0	100.0	100.0	100.0
4	avian	6	98.4	98.8	100.0	100.0
5	avian	4	98.4	98.6	99.9	100.0
5	swine	5	99.3	99.4	100.0	100.0
5,6-like	avian	1	99.9	99.9	99.9	99.9
6	avian	7	94.8	96.2	99.9	100.0
6	swine	1	100.0	100.0	100.0	100.0
7	avian	24	95.7	96.0	99.9	100.0
7	human	1	100.0	100.0	100.0	100.0
7.1	avian	12	98.9	99.0	99.4	99.5
7.2	avian	37	87.2	92.0	98.3	99.9
7.2	swine	1	100.0	100.0	100.0	100.0
9	avian	20	99.7	100.0	100.0	100.0
9	swine	3	100.0	100.0	100.0	100.0
American non-gs/Gd	avian	768	75.4	93.1	99.5	100.0
American non-gs/Gd	swine	2	98.8	98.8	99.2	99.5
Eurasian non-gs/Gd	avian	331	82.7	95.6	100.0	100.0
Eurasian non-gs/Gd	swine	2	100.0	100.0	100.0	100.0
none	avian	48	88.6	91.3	99.7	100.0
