# Supplementary material for: Targeted genomic sequencing of avian influenza viruses in wetland sediment from wild bird habitats
Source: Appl Environ Microbiol. 2024 Jan 23;90(2):e00842-23. doi: 10.1128/aem.00842-23 (PMC10880596; doi:10.1128/aem.00842-23)
Supplement: Table S3 — Probe capture and sequencing metrics. [file aem.00842-23-s0007.pdf]

Table S3: Probe capture and sequencing metrics. Sequencing libraries were prepared from 90 specimens that were positive (n=74) or suspect-positive (n=16) for influenza A virus (IAV) genomic material by RT-qPCR. After pooling libraries together, two independent probe captures were performed and sequenced separately (capture_1 and capture_2). FASTQ data was analyzed with HopDropper to identify distinct fragments of genomic material and remove chimeric artefacts (invalid reads). The number of total read pairs and valid read pairs was reported for each library by HopDropper. Fragment consensus sequences generated by HopDropper were analyzed by FindFlu to identify fragments of IAV genome. The number of IAV read pairs in each library was determined by summing the number of copies for each IAV fragment identified by FindFlu. For each library, the number of IAV read pairs was divided by the total number of read pairs to calculate total on-target rates. Valid on-target rates were calculated by dividing the number of IAV read pairs in a library by the number of valid read pairs in that library.

Capture replicate	Specimen ID	Screening RT-qPCR result	Screening RT-qPCR Ct value	Total read pairs (#)	Valid read pairs (#)	IAV read pairs (#)	Total on-target (%)	Valid on-target (%)
capture-1	sediment-1	Positive	29.74	172839	123424	82372	47.7	66.7
capture-2	sediment-1	Positive	29.74	184143	125720	80859	43.9	64.3
capture-1	sediment-2	Positive	37.80	9816	0	0	0.0	0.0
capture-2	sediment-2	Positive	37.80	13078	0	0	0.0	0.0
capture-1	sediment-3	Positive	34.64	362356	239693	205456	56.7	85.7
capture-2	sediment-3	Positive	34.64	317087	212259	173528	54.7	81.8
capture-1	sediment-4	Positive	37.14	617838	415482	415482	67.2	100.0
capture-2	sediment-4	Positive	37.14	657293	454675	454675	69.2	100.0
capture-1	sediment-5	Positive	34.39	28854	16351	13818	47.9	84.5
capture-2	sediment-5	Positive	34.39	35749	21026	11936	33.4	56.8
capture-1	sediment-6	Positive	35.81	61294	35924	33763	55.1	94.0
capture-2	sediment-6	Positive	35.81	73277	42115	33868	46.2	80.4
capture-1	sediment-7	Positive	37.17	13249	5330	2047	15.5	38.4
capture-2	sediment-7	Positive	37.17	15571	3476	2221	14.3	63.9
capture-1	sediment-8	Positive	37.66	7102	0	0	0.0	0.0
capture-2	sediment-8	Positive	37.66	9909	0	0	0.0	0.0
capture-1	sediment-9	Positive	31.39	220956	150388	110129	49.8	73.2
capture-2	sediment-9	Positive	31.39	187552	129177	95429	50.9	73.9
capture-1	sediment-10	Positive	33.79	59842	37111	15779	26.4	42.5
capture-2	sediment-10	Positive	33.79	76291	46956	20288	26.6	43.2
capture-1	sediment-11	Positive	34.94	40338	21756	7707	19.1	35.4
capture-2	sediment-11	Positive	34.94	71886	43453	31076	43.2	71.5
capture-1	sediment-12	Positive	37.25	30905	16288	10776	34.9	66.2
capture-2	sediment-12	Positive	37.25	40646	21804	13470	33.1	61.8
capture-1	sediment-13	Positive	37.53	530	0	0	0.0	0.0
capture-2	sediment-13	Positive	37.53	642	0	0	0.0	0.0
capture-1	sediment-14	Positive	37.74	6038	1321	781	12.9	59.1
capture-2	sediment-14	Positive	37.74	11660	4015	2628	22.5	65.5
capture-1	sediment-15	Positive	36.97	33403	14578	5164	15.5	35.4
capture-2	sediment-15	Positive	36.97	40416	13794	3763	9.3	27.3
capture-1	sediment-16	Positive	39.90	18514	3857	1853	10.0	48.0
capture-2	sediment-16	Positive	39.90	28731	9055	0	0.0	0.0
capture-1	sediment-17	Positive	36.04	99126	61623	33540	33.8	54.4
capture-2	sediment-17	Positive	36.04	124105	81015	42909	34.6	53.0
capture-1	sedimen-18	Positive	36.91	16335	759	0	0.0	0.0
capture-2	sediment-18	Positive	36.91	21078	0	0	0.0	0.0
capture-1	sediment-19	Positive	38.27	24676	8550	8397	34.0	98.2
capture-2	sediment-19	Positive	38.27	24111	6567	6567	27.2	100.0
capture-1	sediment-20	Positive	39.54	7927	652	0	0.0	0.0
capture-2	sediment-20	Positive	39.54	11547	1809	1809	15.7	100.0
capture-1	sediment-21	Positive	38.93	16819	3886	1191	7.1	30.6

Capture replicate	Specimen ID	Screening RT-qPCR result	Screening RT-qPCR Ct value	Total read pairs (#)	Valid read pairs (#)	IAV read pairs (#)	Total on-target (%)	Valid on-target (%)
capture-2	sediment-21	Positive	38.93	21911	4759	4084	18.6	85.8
capture-1	sediment-22	Positive	38.16	10044	0	0	0.0	0.0
capture-2	sediment-22	Positive	38.16	13722	148	148	1.1	100.0
capture-1	sediment-23	Positive	37.49	28942	15618	9076	31.4	58.1
capture-2	sediment-23	Positive	37.49	34369	18373	7740	22.5	42.1
capture-1	sediment-24	Positive	39.23	192	0	0	0.0	0.0
capture-2	sediment-24	Positive	39.23	205	0	0	0.0	0.0
capture-1	sediment-25	Positive	38.26	18810	9709	2416	12.8	24.9
capture-2	sediment-25	Positive	38.26	11653	3375	33	0.3	1.0
capture-1	sediment-26	Positive	37.92	17050	4401	0	0.0	0.0
capture-2	sediment-26	Positive	37.92	20050	3748	0	0.0	0.0
capture-1	sediment-27	Positive	35.76	48138	25237	9404	19.5	37.3
capture-2	sediment-27	Positive	35.76	51918	25737	6616	12.7	25.7
capture-1	sediment-28	Positive	37.75	42399	20550	13367	31.5	65.0
capture-2	sediment-28	Positive	37.75	45617	19371	9055	19.9	46.7
capture-1	sediment-29	Positive	38.79	19025	4609	4047	21.3	87.8
capture-2	sediment-29	Positive	38.79	23342	3603	529	2.3	14.7
capture-1	sediment-30	Positive	33.48	296600	200680	92475	31.2	46.1
capture-2	sediment-30	Positive	33.48	303294	208434	100689	33.2	48.3
capture-1	sediment-31	Positive	36.04	51796	30680	15993	30.9	52.1
capture-2	sediment-31	Positive	36.04	53945	31069	24441	45.3	78.7
capture-1	sediment-32	Positive	34.38	118096	76146	60574	51.3	79.5
capture-2	sediment-32	Positive	34.38	107319	69349	49813	46.4	71.8
capture-1	sediment-33	Positive	37.00	12889	2179	0	0.0	0.0
capture-2	sediment-33	Positive	37.00	18721	4587	4203	22.5	91.6
capture-1	sediment-34	Positive	38.66	7925	0	0	0.0	0.0
capture-2	sediment-34	Positive	38.66	10580	146	0	0.0	0.0
capture-1	sediment-35	Positive	35.24	96018	57214	35142	36.6	61.4
capture-2	sediment-35	Positive	35.24	99988	49410	29437	29.4	59.6
capture-1	sediment-36	Positive	34.21	318299	207819	106905	33.6	51.4
capture-2	sediment-36	Positive	34.21	297011	189799	81687	27.5	43.0
capture-1	sediment-37	Positive	35.14	59164	30848	11874	20.1	38.5
capture-2	sediment-37	Positive	35.14	59973	31689	17038	28.4	53.8
capture-1	sediment-38	Positive	39.48	57390	23454	17437	30.4	74.3
capture-2	sediment-38	Positive	39.48	56215	20925	7968	14.2	38.1
capture-1	sediment-39	Positive	37.53	14108	0	0	0.0	0.0
capture-2	sediment-39	Positive	37.53	19305	0	0	0.0	0.0
capture-1	sediment-40	Positive	35.43	52572	29150	13058	24.8	44.8
capture-2	sediment-40	Positive	35.43	53874	22204	7616	14.1	34.3
capture-1	sediment-41	Positive	36.85	49970	29206	17826	35.7	61.0
capture-2	sediment-41	Positive	36.85	49103	26999	9784	19.9	36.2
capture-1	sediment-42	Positive	34.88	123004	80528	60880	49.5	75.6
capture-2	sediment-42	Positive	34.88	125657	81782	48570	38.7	59.4
capture-1	sediment-43	Positive	36.67	26365	8973	7365	27.9	82.1
capture-2	sediment-43	Positive	36.67	24860	6651	3996	16.1	60.1
capture-1	sediment-44	Positive	37.97	64481	39264	20760	32.2	52.9
capture-2	sediment-44	Positive	37.97	62189	35994	31542	50.7	87.6
capture-1	sediment-45	Positive	34.01	327306	212395	87909	26.9	41.4
capture-2	sediment-45	Positive	34.01	322004	215898	83987	26.1	38.9
capture-1	sediment-46	Positive	33.75	281204	178445	90685	32.2	50.8
capture-2	sediment-46	Positive	33.75	318599	209473	125384	39.4	59.9
capture-1	sediment-47	Positive	31.00	293899	178571	71589	24.4	40.1

Capture replicate	Specimen ID	Screening RT-qPCR result	Screening RT-qPCR Ct value	Total read pairs (#)	Valid read pairs (#)	IAV read pairs (#)	Total on-target (%)	Valid on-target (%)
capture-2	sediment-47	Positive	31.00	335264	205977	54692	16.3	26.6
capture-1	sediment-48	Positive	37.37	12068	1220	0	0.0	0.0
capture-2	sediment-48	Positive	37.37	15492	1243	0	0.0	0.0
capture-1	sediment-49	Positive	35.74	20204	6623	750	3.7	11.3
capture-2	sediment-49	Positive	35.74	25349	6566	3786	14.9	57.7
capture-1	sediment-50	Positive	30.51	434665	265394	122267	28.1	46.1
capture-2	sediment-50	Positive	30.51	399178	263511	111366	27.9	42.3
capture-1	sediment-51	Positive	38.78	32628	14046	10786	33.1	76.8
capture-2	sediment-51	Positive	38.78	45528	21216	17323	38.0	81.7
capture-1	sediment-52	Positive	27.86	2845588	1869576	1163089	40.9	62.2
capture-2	sediment-52	Positive	27.86	2879923	1924536	1195950	41.5	62.1
capture-1	sediment-53	Positive	37.85	12915	3572	2756	21.3	77.2
capture-2	sediment-53	Positive	37.85	17538	3878	1405	8.0	36.2
capture-1	sediment-54	Positive	38.26	70353	47014	37520	53.3	79.8
capture-2	sediment-54	Positive	38.26	101699	67246	53801	52.9	80.0
capture-1	sediment-55	Positive	37.32	24	0	0	0.0	0.0
capture-2	sediment-55	Positive	37.32	20	0	0	0.0	0.0
capture-1	sediment-56	Positive	39.89	8834	0	0	0.0	0.0
capture-2	sediment-56	Positive	39.89	11661	0	0	0.0	0.0
capture-1	sediment-57	Positive	36.32	21516	0	0	0.0	0.0
capture-2	sediment-57	Positive	36.32	31734	7919	7919	25.0	100.0
capture-1	sediment-58	Positive	36.83	35728	15287	3075	8.6	20.1
capture-2	sediment-58	Positive	36.83	32223	12298	4398	13.6	35.8
capture-1	sediment-59	Positive	33.37	9519	999	493	5.2	49.3
capture-2	sediment-59	Positive	33.37	11220	161	0	0.0	0.0
capture-1	sediment-60	Positive	37.16	3027	0	0	0.0	0.0
capture-2	sediment-60	Positive	37.16	4094	0	0	0.0	0.0
capture-1	sediment-61	Positive	38.36	6825	2150	2150	31.5	100.0
capture-2	sediment-61	Positive	38.36	9139	2801	2801	30.6	100.0
capture-1	sediment-62	Positive	32.11	290124	187124	156618	54.0	83.7
capture-2	sediment-62	Positive	32.11	304333	208477	152573	50.1	73.2
capture-1	sediment-63	Positive	32.18	202819	124051	83566	41.2	67.4
capture-2	sediment-63	Positive	32.18	196436	126576	88699	45.2	70.1
capture-1	sediment-64	Positive	35.26	1325	0	0	0.0	0.0
capture-2	sediment-64	Positive	35.26	1700	0	0	0.0	0.0
capture-1	sediment-65	Positive	35.40	50031	25932	18793	37.6	72.5
capture-2	sediment-65	Positive	35.40	43528	17321	12025	27.6	69.4
capture-1	sediment-66	Positive	36.38	4304	0	0	0.0	0.0
capture-2	sediment-66	Positive	36.38	5579	0	0	0.0	0.0
capture-1	sediment-67	Positive	38.45	70801	46010	18791	26.5	40.8
capture-2	sediment-67	Positive	38.45	78229	52760	16894	21.6	32.0
capture-1	sediment-68	Positive	38.62	31988	13962	8058	25.2	57.7
capture-2	sediment-68	Positive	38.62	28440	10387	3771	13.3	36.3
capture-1	sediment-69	Positive	34.14	327166	198942	133144	40.7	66.9
capture-2	sediment-69	Positive	34.14	366586	233981	151472	41.3	64.7
capture-1	sediment-70	Positive	36.99	3377	0	0	0.0	0.0
capture-2	sediment-70	Positive	36.99	4653	0	0	0.0	0.0
capture-1	sediment-71	Positive	33.89	132503	86232	66531	50.2	77.2
capture-2	sediment-71	Positive	33.89	136011	87949	74312	54.6	84.5
capture-1	sediment-72	Positive	36.30	84741	57300	57300	67.6	100.0
capture-2	sediment-72	Positive	36.30	84098	54494	36013	42.8	66.1
capture-1	sediment-73	Positive	37.02	32700	20535	4581	14.0	22.3

Capture replicate	Specimen ID	Screening RT-qPCR result	Screening RT-qPCR Ct value	Total read pairs (#)	Valid read pairs (#)	IAV read pairs (#)	Total on-target (%)	Valid on-target (%)
capture-2	sediment-73	Positive	37.02	32030	19401	7514	23.5	38.7
capture-1	sediment-74	Positive	36.36	3011	0	0	0.0	0.0
capture-2	sediment-74	Positive	36.36	3948	0	0	0.0	0.0
capture-1	sediment-75	Suspect		42	0	0	0.0	0.0
capture-2	sediment-75	Suspect		30	0	0	0.0	0.0
capture-1	sediment-76	Suspect		110	0	0	0.0	0.0
capture-2	sediment-76	Suspect		83	0	0	0.0	0.0
capture-1	sediment-77	Suspect		5154	0	0	0.0	0.0
capture-2	sediment-77	Suspect		6996	0	0	0.0	0.0
capture-1	sediment-78	Suspect		4327	0	0	0.0	0.0
capture-2	sediment-78	Suspect		6691	681	0	0.0	0.0
capture-1	sediment-79	Suspect		22	0	0	0.0	0.0
capture-2	sediment-79	Suspect		23	0	0	0.0	0.0
capture-1	sediment-80	Suspect		24	0	0	0.0	0.0
capture-2	sediment-80	Suspect		11	0	0	0.0	0.0
capture-1	sediment-81	Suspect		8203	0	0	0.0	0.0
capture-2	sediment-81	Suspect		11354	0	0	0.0	0.0
capture-1	sediment-82	Suspect	41.08	3450	248	0	0.0	0.0
capture-2	sediment-82	Suspect	41.08	5799	1158	295	5.1	25.5
capture-1	sediment-83	Suspect		9056	0	0	0.0	0.0
capture-2	sediment-83	Suspect		12411	0	0	0.0	0.0
capture-1	sediment-84	Suspect	40.83	6461	705	705	10.9	100.0
capture-2	sediment-84	Suspect	40.83	7461	634	634	8.5	100.0
capture-1	sediment-85	Suspect		12275	4537	4537	37.0	100.0
capture-2	sediment-85	Suspect		15046	5034	5034	33.5	100.0
capture-1	sediment-86	Suspect		19177	7919	2106	11.0	26.6
capture-2	sediment-86	Suspect		17940	5936	661	3.7	11.1
capture-1	sediment-87	Suspect		3038	0	0	0.0	0.0
capture-2	sediment-87	Suspect		4335	0	0	0.0	0.0
capture-1	sediment-88	Suspect		22078	8490	8490	38.5	100.0
capture-2	sediment-88	Suspect		20958	4620	4620	22.0	100.0
capture-1	sediment-89	Suspect		18321	7915	4114	22.5	52.0
capture-2	sediment-89	Suspect		22887	8962	3379	14.8	37.7
capture-1	sediment-90	Suspect		8826	201	201	2.3	100.0
capture-2	sediment-90	Suspect		12281	403	403	3.3	100.0
capture-1	Undetermined			987909	30901	0	0.0	0.0
capture-2	Undetermined			989527	40473	0	0.0	0.0
capture-1	control-1			1727947	25754	0	0.0	0.0
capture-2	control-1			1697915	21957	0	0.0	0.0
capture-1	control-2			1251991	21795	0	0.0	0.0
capture-2	control-2			1219539	9900	0	0.0	0.0
capture-1	control-3			862722	2003	0	0.0	0.0
capture-2	control-3			877188	2514	0	0.0	0.0
capture-1	control-4			2156783	4287	0	0.0	0.0
capture-2	control-4			2115832	6104	0	0.0	0.0
capture-1	control-5			1277530	18481	0	0.0	0.0
capture-2	control-5			1302672	19157	0	0.0	0.0
capture-1	control-6			1596828	1234	0	0.0	0.0
capture-2	control-6			1577998	0	0	0.0	0.0
